# Erythrocyte sphingosine kinase regulates intraerythrocytic development of *Plasmodium falciparum*

**DOI:** 10.1038/s41598-020-80658-7

**Published:** 2021-01-13

**Authors:** Raj Kumar Sah, Soumya Pati, Monika Saini, Shailja Singh

**Affiliations:** 1 Special Centre for Molecular Medicine, Jawaharlal Nehru University, New Delhi, India; 2 Department of Life Sciences, School of Natural Sciences, Shiv Nadar University, Greater Noida, India

**Keywords:** Cell growth, Cell signalling, Cellular imaging, Glycobiology, Mechanisms of disease, Drug discovery, Drug screening, Target identification, Malaria

## Abstract

The sphingolipid pool is key regulator of vital cellular functions in *Plasmodium falciparum* a causative agent for deadly malaria. Erythrocytes, the host for asexual stage of *Plasmodium*, are major reservoir for Sphingosine-1-phosphate (S1P). Erythrocyte possesses Sphingosine kinase (SphK) that catalyzed its biosynthesis from sphingosine (Sph). Since, *Plasmodium* lacks SphK homologous protein it can be envisaged that it co-opts sphingolipids from both intraerythrocytic as well as extracellular pools for its growth and development. Herein, by sphingosine-NBD probing, we report that infected erythrocytes imports Sph from extracellular pool, which is converted to S1P and thereby taken by *P. falciparum*. Next, by targeting of the SphK through specific inhibitor N,N-Dimethylsphingosine DMS, we show a reduction in erythrocyte endogenous S1P pool and SphK-phosphorylation that led to inhibition in growth and development of ring stage *P. falciparum.* Owing to the role of S1P in erythrocyte glycolysis we analyzed uptake of NBD-Glucose and production of lactate in DMS treated and untreated plasmodium. DMS treatment led to decreased glycolysis in *Plasmodium*. Interestingly the host free *Plasmodium* did not show any effect on glycolysis with DMS treatment indicating its host-mediated effect. Further to understand the *in-vivo* anti-plasmodial effects of exogenous and endogenous erythrocyte S1P level, Sphingosine-1-phosphate lyase (S1PL) inhibitor (THI), S1P and SphK-1 inhibitor (DMS), were used in *Plasmodium berghei* ANKA (PbA) mice model. DMS treatment led to reduction of endogenous S1P conferred significant decrease in parasite load, whereas the plasma level S1P modulated by (THI) and exogenous S1P have no effect on growth of *Plasmodium*. This suggested erythrocyte endogenous S1P pool is important for *Plasmodium* growth whereas the plasma level S1P has no effect. Altogether, this study provides insight on cellular processes regulated by S1P in *P. falciparum* and highlights the novel mechanistically distinct molecular target i.e. SphK-1.

## Introduction

An estimate of 219 million malaria cases and 435,000 related deaths were reported by World Health Organization (WHO, 2018). The limitable anti-malarial drugs and emergence of parasitic resistance to almost every available chemotherapeutics continues to spur the search for novel approaches. Though the next generation of anti-malarial targeting the novel parasite encoded enzymes and molecular pathways look promising^1,2^ but they are vulnerable to develop resistance because of their constant regulation by parasite genome by default^3,4^. To deal with the nuisance of drug resistance, targeting host encoded proteins that are indispensable for parasite growth and survival would be an ideal situation. Since, the chances of development of resistance against non-parasite targets are very bleak, host targeted drug development will be a classical addition to the field of drug discovery in malaria. Emerging studies highlighted that malaria parasite scavenges many of its critical metabolites from the host erythrocytes for its intracellular growth during its asexual blood stage (ABS)^5^. Among these, Plasmodium has shown to be strictly co-dependent upon host-secreted lipid metabolic pool^6^. For example, phosphatidylcholine (PC) is derived primarily from choline, which is supplied in serum containing lysophosphatidylcholine. In the lack of choline, both ethanolamine and serine are used by the parasite. Phosphatidylethanolamine (PE) is synthesized almost equally with ethanolamine and serine and can be substituted for each other by both precursors. Serine in and phosphatidylserine (PS) comes primarily from host cell hemoglobin breakdown via the parasite. This information suggests extraordinary flexibility of *P. falciparum* towards host-derived phospholipids (PL)^7^. Interestingly, compounds targeting membrane lipids like, PC, PE or phosphatidylinositol (PI) 4-phosphate have shown potent antimalarial activity earlier because of their synthesis, uptake and transport are essential for the viability of *P. falciparum*^8–10^. When *P. falciparum* invades the host, there is marked reduction in the level of sphingolipid was observed on its surface, indicating a possible parasite-dependent internalization or degradation of sphingomyelin^11^. In addition, during invasion, *plasmodium* generates new membrane forms along with parasitophorous vacuole, and then spans the host cytoplasm to construct new trafficking networks essential for smooth functioning of import and export system, which is responsible for parasite growth and multiplication. Notably, use of Sphingolipid analogues, such as; fumonisin B1 or 1-phenyl-2-palmitoylamino-3-morpholino-1-propanol (PPMP) and 1-deoxysphingoid bases have shown promising antimalarial activity by strongly intervening plasmodial sphingolipid metabolism^12^. Notably, antimalarials like mefloquine and artemisinin have shown to increase the intracellular levels of ceramide in infected erythrocytes that lead to depletion of parasite glutathione, thereby preventing the degradation of toxic ferriprotoporphyrin IX (FP) in parasites following its accumulation^13^. Interestingly, decreased serum S1P concentration has been associated with malaria patient infected with *P. vivax* and *P. falciparum*, suggesting involvement of S1P signaling cascade in the severity of malaria^14^. It is noteworthy that, S1P, a pleotropic signaling sphingolipid is catalyzed by the sphingosine kinases (SphK) that contributes in critical biological processes involving; cell proliferation and survival, endothelial cell migration, maintenance of endothelial barrier integrity and bone marrow trafficking etc.^15,16^. The *plasmodium* sphingolipid metabolic pathway is known to have activity of several enzymes (e.g., sphingomyelin synthase, glucosylceramide synthase, and two sphingomyelinases)^11,17–19^. Lipids enriched in the host cell may serve as a reservoir for parasite-mediated salvage. Previous studies have shown that *P. falciparum* may import certain lipid species such as ceramides, sphingolipids and Lyso PCs^20–22^. However, *Plasmodium* does not have any form of SphK that would synthesize S1P from it precursor sphingosine (Sph). Thus, we hypothesized that *P. falciparum* might scavenge the sphingosine metabolites for its intracellular growth during ABS. Plausibly, the parasite-infected erythrocytes utilize glucose at a much higher rate than the normal parasite uninfected erythrocytes and lacks a functional tricarboxylic acid (TCA) cycle. Thus parasite completely depends upon pre-existing pools of host glucose as well as imports it by expressing glucose transporters on the surface of the host erythrocytes^23–25^. The parasite’s sole dependency on glycolysis for energy needs makes it a potential target for anti-malarial chemotherapies. However, the regulation of glycolysis by parasites in infected erythrocytes is not very clear. A recent study demonstrated that intracellular S1P facilitates glycolysis in erythrocytes in response to hypoxia sequentially via; (i) binding to deoxygenated- hemoglobin, and (ii) translocating it to the membrane followed by releasing of glycolytic enzyme, GAPDH that regulates glycolysis in host erythrocytes^26^. Since parasite lacks Sph to S1P converting enzyme and mitochondrial ATP synthesis pathway^27^ thus it relies mainly on glycolysis for its energy supply during ABS^28^. Thus, we have tried to elucidate the impact of host-S1P and host-glycolysis pathways and their possible crosstalk in infected erythrocytes, and their impact on overall parasite growth and multiplication. In this study, we have deciphered three key findings, such as; (i) *P. falciparum* strictly depends upon S1P, an intermediate of host sphingolipid pathway for its intracellular growth during ABS, (ii) blocking of host-SphK-1 by inhibitor DMS led to depleted S1P, followed by translocation of GAPDH from cytosol to membrane, resulting in altered glycolysis and (iii) blocking SphK-1 activity in host-free parasites revealed no change in lactate level, suggesting erythrocytes as the mediator of S1P-dependent glycolysis in *P. falciparum* infected erythrocytes. Overall, our study unravelled molecular insights of exploitable host-dependent metabolic circuits by *P. falciparum* that can aid in designing of novel antimalarials in future.

## Materials and methods

### Cultivation of *P. falciparum-*infected erythrocytes

*P. falciparum* 3D7 strain was cultured in RPMI 1640 (Invitrogen, Carlsbad, CA, USA) supplemented with 27.2 mg/L hypoxanthine (Sigma-Aldrich, St. Louis, MO, USA), 2 gm/L sodium bicarbonate (Sigma-Aldrich, St. Louis, MO, USA) and 0.5 gm/L AlbuMax I (Gibco, Grand Island, NY, USA) using O+ human erythrocytes, under mixed gas environment (5% O_2_, 5% CO_2_ and 90% N_2_) as described previously^29^. For the assessment of half-maximal drug concentration for inhibition of malaria survival (IC_50_) values, synchronized *P. falciparum* infected erythrocyte cultures were used at late-ring or early trophozoite stage (18–24 h post infection (hpi)) at a parasitemia of 1%. Where indicated, cultures were treated with the N,N-Dimethylsphingosine (DMS) inhibitor (Sigma-Aldrich, St. Louis, MO, USA) at concentrations ranging from 0 to 40 µM. Untreated controls were cultured in parallel under the same conditions and processed identically. To assess total parasitemia, cultures were collected at the indicated times and were lysed by freeze-thawed. The lysates were processed for SYBR-green staining (Thermo Fisher Scientific, Waltham, Massachusetts, US). Briefly, equal volumes of lysis buffer containing 20 mM tris (pH 7.5), 5 mM EDTA, 0.008% saponin (*w/v*), and 0.08% triton x-100 (*v*/*v*) was added to the lysed parasites and incubated for 3 h at 37 °C with 1 × SYBR-green dye. Fluorescence after the SYBR-green assay was recorded in a multimode plate reader (Bio‐Rad) at an excitation and emission wavelength of 485 nm and 530 nm, respectively. Percent growth inhibition was calculated using the following formula: % Growth Inhibition = {(Control − Treated)/ (Control) * 100}. The effect of DMS was tested on progression of the parasites treated at ring stage and monitored until different developmental asexual stages, namely, rings, trophozoites, schizonts and release of merozoites from schizonts. Tightly synchronized ring stage parasite cultures treated with 14 μM DMS inhibitor or solvent as control were incubated for 0, 18, 34, 45 h blood stage asexual cycle to monitor the progression at each stage. Morphological analysis and counting (~ 3,000 cells/ Giemsa-stained slides in duplicate) were done at each of these stages to monitor the parasite’s progression.

### Analysis of morphological changes in infected and uninfected erythrocytes by scanning electron microscopy (SEM) after DMS treatment

The infected and uninfected erythrocytes were treated with DMS (14 µM) for a period of 5 h. Following incubation erythrocyte and infected erythrocyte were washed three times in sterile PBS. The samples were then fixed by 2.5% glutaraldehyde in 1 × PBS (pH 7.4) with 2% formaldehyde for a period of 30 min. Post fixation, samples were rinsed thrice with 1 × PBS and dehydrated in absolute ethanol series (ethanolic dehydration), using a standard protocol. Samples were then completely dried, coated with gold, and observed under the scanning electron microscope.

### Extraction of lipids and sphingosine-1-phosphate measurement

Equal number of synchronous parasitized erythrocytes at 7–8% parasitemia, and uninfected erythrocytes with 2.6 × 10^9^ cells/ 200 µl of packed volume both in presence and absence of DMS for a period of 5 h were used for further experiments. Collected cell pellets and supernatant were employed for lipid extraction as reported previously^30^. Pellets were resuspended in 100 µl H_2_O and transferred to 900 µl methanol containing S1P (S9666, Sigma-Aldrich) 200 nM as internal standard. Whereas, for quantification of S1P in supernatant, 1:15 ratio of methanol containing S1P (200 nM) as internal standard was used. After vortexing and centrifugation at 10,000×*g* for 5 min at RT, methanol extracts were removed to a new glass tube. After evaporation by N_2_, dried lipids were resuspended in 200 µl methanol. Extracted lipid samples were subjected to liquid-chromatography mass-spectrometry (LC/MS) analysis. We used a Waters Acquity H-Class UPLC-system (Waters, Milford, MA, USA). Chromatographic separation was achieved on an Acquity BEH C18, 1.7 μm, 75 × 2.1 mm column (Waters, Manchester, UK). Mass spectrometry was performed in negative electrospray mode using a high-resolution mass spectrometer synapt G2 S HDMS (Waters, Manchester, UK) with a TOF-detector with linear dynamic range of at least 5000:1. The mass spectra were acquired over the range of 100–1000 Da with a spectral acquisition rate of 0.1 s per spectrum.

For S1P measurement by enzyme-linked immunosorbent assay (ELISA) (My BioSource, San Diego, USA) method, parasite infected and uninfected erythrocytes were treated with 14 μM DMS for 5 h at 37 °C. Collected supernatant was used to measure extracellular S1P, whereas, the lysed erythrocytes were used to measure intracellular S1P level. The samples were added to the micro ELISA plate wells separately, pre-coated with S1P-specific antibody for 90 min at 37 °C followed by probing with a biotinylated detection antibody specific to human S1P and incubation for 1 h at 37 °C. After washing, Avidin-horseradish peroxidase (HRP) conjugate was added successively to each microplate well and incubated for 30 min at 37 °C. The wells were washed to remove the unbound components followed by addition of substrate solution to each well. Only those wells that contained S1P, biotinylated detection antibody and Avidin-HRP conjugate appeared blue in color. The enzyme–substrate reaction was terminated by addition of stop solution turning the reaction color to yellow. The optical density (O.D.) proportional to the S1P level was measured spectrophotometrically at a wavelength of 450 nm.

### Fluorescence microscopy for uptake of NBD-sphingosine in infected erythrocyte

Infected erythrocytes (1 × 10^8^/mL) of trophozoite stage were washed and incubated with 1 μM omega (7-nitro-2–1, 3-benzoxadiazol-4-yl) (2S,3R,4E)-2-amino octadec-4- ene-1,3-diol (NBD–sphingosine; Avanti Polar Lipids) for 15 min at 37 °C. The infected erythrocytes were pelleted at 550×*g* for 5 min and resuspended in fresh incomplete RPMI media with 0.1% BSA. Approximately 100 μL of the samples was placed onto a glass bottom petri dish. The cells were then allowed to settle at RT for 5 min and were viewed using a confocal Nikon Ti2 microscope equipped with a 100 × oil objective (Melville, NY). Digital images were captured. Further, images were processed via NIS-Elements software.

### Quantification of NBD-S1P with SphK-1 inhibitor using microplate reader

For the fluorometric SPHK assay, fluorescent substrate was used and the conversion of NBD-sphingosine (NBD-Sph) to NBD-S1P in uninfected, infected erythrocyte was quantified both in post treatment host free parasite and host free parasite pre-treatment. 100 µl of uninfected and infected erythrocyte suspension (1 × 10^9^ cells) with 6 to 8% parasitemia in iRPMI media and 100 µl of iRPMI containing 10 µM NBD-Sph were incubated at 37 °C for 10 min. Then, the erythrocytes and the media containing NBD-Sph were mixed (final NBD-Sph concentration: 5 µM) and incubated at 37 °C for 60 min. After incubation, lipids were extracted from 200 µl of the media containing uninfected and infected erythrocytes and saponin lysed parasites. Then, 260 µl of methanol and 400 µl of chloroform/methanol (1:1) were added to the samples and thoroughly mixed. Subsequently, 16 µl of 7 M NH4OH, 400 µl of chloroform, and 300 µl of 1.5 M KCl were added to the samples and mixed thoroughly. The lipids were separated by centrifugation for 5 min at 17,000×*g*^31^ and 100 μl of the upper (aqueous) phase transferred to a black 96-well plate. The fluorescence intensities of the upper aqueous phases containing NBD-S1P were measured at 530 nm with excitation at 485 nm in 96-well plates using varioskan LUX Multimode Microplate Reader (Thermo fisher, Massachusetts, USA).

### Immunofluorescence assay and immunoblotting for SphK-1 and GAPDH in erythrocytes

For immunofluorescence assays, thin smears of schizont or mixed stage parasites treated or untreated with DMS were made on glass slides, air dried and fixed with methanol (ice cold) for 30 min at − 20 °C. Smears were blocked with 3% (*w*/*v*) bovine serum albumin (BSA) in phosphate buffer saline (PBS) containing blocking buffer (pH 7.4) for 30 min at room temperature (RT). Slides were probed with anti-SphK-1 (Invitrogen, Carlsbad, CA, USA, 1:1000) rabbit and anti-GAPDH (Invitrogen, Carlsbad, CA, USA, 1:500) mouse antibodies in blocking buffer at RT for 1 h. After washing, slides were incubated with Alexa Fluor 594 conjugated goat anti-rabbit IgG (Molecular Probes, USA, 1:500) and Alexa Fluor 488 conjugated goat anti-mouse IgG (Molecular Probes, USA, 1:500) at RT for 1 h. After washing, the slides were mounted in ProLong Gold antifade reagent (Invitrogen, Carlsbad, CA, USA), viewed on a Nikon A1-R confocal microscope and Olympus confocal microscopy. Further, images were processed via NIS-Elements software.

### Immunoblotting for SphK-1 and GAPDH in erythrocytes

Uninfected erythrocytes and parasite infected erythrocytes in the presence or absence of DMS were lysed by freeze-thawing in 10 volumes of 5 mmol/l cold phosphate buffer (pH 8.0) with 1 × protease inhibitor cocktail (PIC) and vortexed to isolate erythrocyte membrane. Next, to isolate erythrocyte membrane, all samples were centrifuged at 3200×*g* for isolation of supernatant devoid of parasites followed by centrifugation of supernatant at 20,000×*g* for 20 min at 4 °C to isolate erythrocyte membrane pellets . The membrane pellets were washed ten times with phosphate buffer to obtain ghost erythrocytes. Total cell pellet of parasite infected and uninfected erythrocytes were re-suspended in RIPA buffer [100 mM phosphate buffer pH 7.2, 150 mM NaCl, 1% NP-40, 0.5% sodium deoxycholate, 0.1% SDS, 50 mM EDTA and 1 × PIC. Equal amounts of each sample were boiled with 2 × Laemmli buffer, separated on a 10% polyacrylamide gel, transferred onto the PVDF membranes (Millipore) followed by blocking with 5% skim milk blocking buffer for 1 h at 4 °C. After washing, blots were incubated for 1 h with anti-SphK-1 (1:3000), anti-Phospho-SphK-1 (Ser225) (Invitrogen, Carlsbad, CA, USA, 1:1000) rabbit and anti-GAPDH (Invitrogen, Carlsbad, CA, USA, 1:10,000) mouse antibodies in blocking buffer. Later, the blots were washed and incubated for 1 h with appropriate secondary anti-rabbit and anti-mouse (1:10,000) antibodies conjugated to HRP. Immunoblotted proteins were visualized by using the Clarity Western ECL substrate (Bio-Rad).

### Glycolysis estimation by measurement of lactate level and glucose uptake

Glycolysis estimation was performed using lactate assay kit (MAK064-1KT, Sigma-Aldrich, St. Louis, MO, USA). Parasite infected and uninfected erythrocytes were resuspended in cRPMI containing 10 µM DMS and incubated for 3 h at 37 °C. The supernatant was separated by centrifugation at 5000×*g* for 10 min at RT and lactate levels were measured in supernatant and lysed erythrocytes according to the manufacturer's protocol. Glucose uptake into the parasites was quantified with 2-(*N*-(7 Nitrobenz-2-oxa-1, 3-diazol-4-yl) Amino)-2-Deoxyglucose (2-NBDG), a fluorescent D‐glucose derivative-based tracer (Sigma-Aldrich, St. Louis, MO, USA) via fluorescence labelling and flow cytometry. Synchronized trophozoite stage parasites were treated with 10 μM DMS and incubated for 3 h at 37 °C. The parasite medium was replaced with RPMI without glucose supplementation with 0.3 mM 2‐NBDG, followed by incubation for 20 min at 37 °C to allow the uptake of the glucose analogue. The 2-NBDG uptake reaction was stopped by withdrawing the incubation medium and washing the cells twice with RPMI media followed by phosphate buffered saline (PBS). Cells were viewed on a Nikon A1-R confocal microscope (Nikon, Tokyo, Japan) and further, images were processed via NIS-Elements software. Further same samples were used to measure fluorescence intensity of 2-NBDG labelled cells by flow cytometry (FACS-LSR Fortessa; BD Biosciences). BD FACS-DIVA Software was used to acquire the cells, for each measurement. Data from 1 × 10^6^ single-cell events were collected and data analysis was performed using FlowJo software (Tree Star).

### Animal handling

Animal studies were performed in accordance with guidelines of the Institutional Animal Ethics Committee (IEAC) of Jawaharlal Nehru University (JNU), Delhi and Committee for Control and Supervision of Experiments on Animals (CPCSEA). The protocols for animal experiments and usage of laboratory animals were approved under strict accordance of ethical guidelines, approved by the animal ethics committee IAEC-JNU. Mice obtained from Central Laboratory Animal Resources, Jawaharlal Nehru University, Delhi were housed under standard conditions of food, temperature (25 °C ± 3), relative humidity (55 ± 10%) and illumination (12 h light/dark cycles) throughout the experiment.

### Infection of *Plasmodium berghei* ANKA (PbA) in BALB/c mice

Chloroquine-sensitive *Plasmodium berghie* ANKA strain (PbA) was used for the induction of malaria in the experimental BALB/c mice. Mice infected with PbA were used as the donor source. The donor infected mice with a parasitemia of 20–30% were sacrificed, and blood was collected by cardiac puncture into heparinized tubes. The blood was subsequently diluted with 0.9% normal saline solution and the infection was induced by injecting 0.2 ml of the diluted blood containing 1 × 10^8^ parasitized erythrocytes via IP injection^32^. Parasitemia was monitored daily by microscopic examination of Giemsa stained thin blood smears, and was calculated using the following formula, % parasitemia = Number of parasitized erythrocytes × 100/ Total number of erythrocytes.

### Evaluation of in vivo antimalarial therapeutic efficacy

Evaluation of the antimalarial potential of DMS, THI, S1P was performed. At day 2 after infection, tail blood samples were taken for determination of parasitemia before administration of test compounds. DMS and 2-Acetyl-4-tetrahydroxybutyl imidazole (THI) (4 µg/kg) and S1P (0.4 µg/kg) were administered using intraperitoneal, oral and intravenous routes of administrations respectively^33–35^^.^ Each group of mice received the first dose of test compound on day 2 followed by further administration once a day for 3 consecutive days (day 0–3). Tail blood samples for determination of parasitemia were taken on day 3, i.e. 24 h after the first treatment dose. Untreated control mice were given 10 ml/kg of distilled water (DW). Parasitemia was monitored daily by microscopic examination of Giemsa stained thin blood smears and calculated using the formula. % parasitemia = Number of parasitized erythrocytes × 100/ Total number of erythrocytes.

### Statistical analysis

The data for all the assays are expressed as the mean ± standard deviation (SD) of three independent experiments done in triplicates. Student’s t-test was performed to calculate the p values, where p < 0.05 was taken as significant. One-way ANOVA along with Tukey’s multiple comparisons test was performed for comparing groups, where *p* < 0.05 was taken as significant.

The data for all the assays are expressed as the mean ± standard deviation (SD) of three independent experiments done in triplicates. Student’s t-test was performed to calculate the p values, where p < 0.05 was taken as significant. One-way ANOVA along with Tukey’s multiple comparisons test was performed for comparing groups, where *p* < 0.05 was taken as significant.

## Results

### Inhibition of host SphK-1 activity by specific inhibitor DMS led to decreased S1P levels in both uninfected and infected erythrocytes

**Figure 1:**
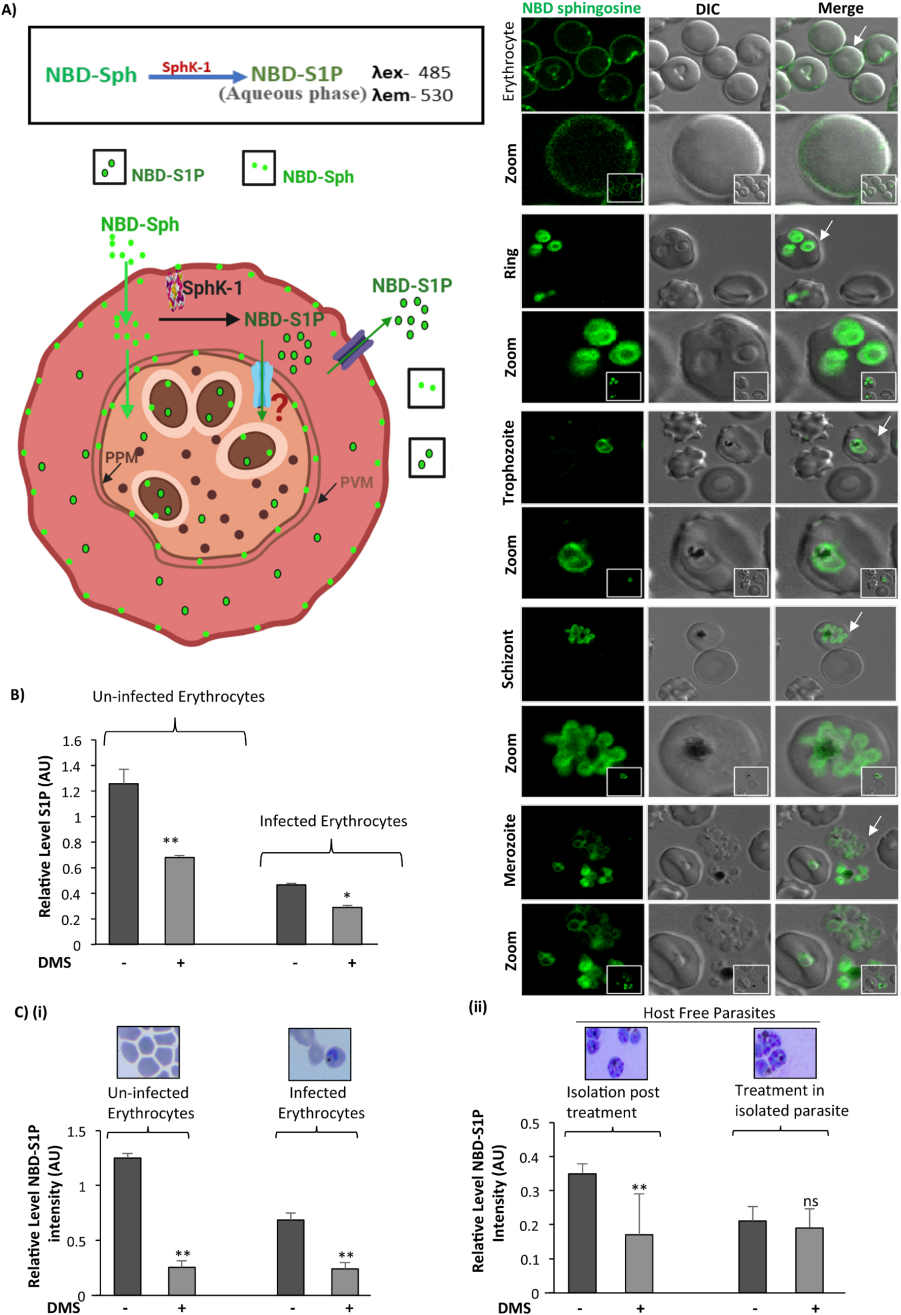
Inhibition of host SphK-1 by specific inhibitor DMS causes decrease in S1P level. (**A**) NBD-Sph and NBD-S1P are localized to host membrane and parasite. Infected erythrocytes was resuspended in buffer containing fatty acid-free BSA (0.1% (w/v)) and incubated with NBD-sphingosine (1 μM) for at least 15 min at 37 °C. The Infected erythrocytes were imaged by fluorescence using a 100 × oil objective on a Nikon Ti2 microscope. Schematic illustration of S1P and NBD-S1P synthesis and uptake in parasite. Erythrocytes incorporate NBD-sphingosine (NBD-Sph), which are phosphorylated by Sphingosine kinase (SphK-1) to NBD-S1P, respectively. Parasites uptake NBD-S1P from erythrocytes. White arrow indicated the erythrocyte chosen for better depiction of NBD-S1P level as in form of zoomed-in images. (**B**) Bar graph depicts ELISA-based S1P quantification in infected and uninfected erythrocytes. (**C** **(i)**) Bar graph denotes the relative level of NBD-S1P. Uninfected erythrocytes, infected erythrocyte pre-treated with DMS were mixed with NBD-sphingosine (NBD-Sph) and incubated for the 1 h at 37 degree. For NBD-S1P quantification, the supernatant and cell pellets were transferred to 1.5-mL tubes containing methanol/chloroform (MeOH/CHCl3) mixture. NH4OH solution and CHCl3 were added to this mixture in a stepwise manner. After centrifugation, NBD-S1P and NBD-sphingosine were separated into aqueous (upper) and organic (lower) phases in the alkaline condition, respectively. The aqueous phases were transferred to a black 96-well plate and the fluorescence of the sample was measured. (**C** **(ii)**) Bar graph denotes the relative level of NBD-S1P in host free parasite post treated and host free parasites pre-treated with DMS, mixed with NBD-sphingosine (NBD-Sph) and incubated for the 1 h at 37 degree Celsius. Giemsa stained image depict the Uninfected erythrocytes and infected erythrocytes of asexual blood stage (ABS) and saponin lysed/Host free parasites. Three independent experiment have been performed, n = 3, ∗*p* ≤ 0.05.

It has been stated previously that the de-novo sphingolipid synthesis occurs in the intraerythrocytic stages of human malaria parasite *P. falciparum* and can be inhibited by well-established ceramide biosynthesis inhibitors^12^. Previous reports suggested that protozoa and fungal pathogens utilize sphingolipid metabolites during host pathogen interactions^17,36,37^. Since *P. falciparum* do not harbor any form of SphK enzymes that convert sphingosine to sphingosine-1-phosphate, we assume that parasite might co-depend upon host derived sphingolipid pathway intermediates for its intracellular growth and proliferation. Since, erythrocytes uptake sphingosine from extracellular sources and convert it to S1P by SphK-1, we asked that, whether the metabolic precursor of S1P, sphingosine gets incorporated during *P. falciparum* infection. To understand this, we have used sphingosine-specific metabolic probe (NBD-Sph) to evaluate its incorporation during infection using fluorescent imaging. The results revealed clear uptake of NBD-Sph in parasites of infected erythrocytes as well as in uninfected erythrocytes. (Fig. 1A). Then we validated the intracellular level of S1P in both infected and uninfected erythrocytes in presence of DMS that inhibits the activity of SphK-1, using ELISA and LC-MS/MS based tools. The findings from ELISA revealed ~ 50% reduction in S1P levels in DMS-treated infected erythrocytes, as compared to untreated infected erythrocytes (Fig. 1B). In parallel, LC/MS analysis of lipid extracts prepared from *P. falciparum* infected and/or uninfected erythrocytes in the presence of DMS, demonstrated a characteristic peak at m/z 378 corresponding to the ion of (S1P) in MS spectra for S1P in all the experiments. The S1P levels in lipid extracts of DMS-treated infected erythrocytes and uninfected erythrocyte were drastically reduced as compared to untreated samples (Supplementary Figs. 1, 2 (A–H)). Further, we evaluated if blocking the host-SphK-1 has any impact on S1P formation in infected erythrocytes by using NBD-Sph labeled probe. This observation suggested that, DMS treatment blocked the S1P formation in both uninfected and infected erythrocytes, as evident by diminished intensities of fluorescent labeled NBD-S1P in fluorometric analysis. Relative level of NBD-S1P is compared within un-infected erythrocyte and infected erythrocyte in presence of DMS (Fig. 1C (i)).

Further, we checked if parasite has any de novo pathway for production of S1P or it opts for host-derived S1P for its own multiplication and development in ABS. To answer this, we have used two experimental conditions for estimating NBD-S1P level in host free parasites; which included, (i) estimation of NBD-S1P in host free parasites isolated from DMS treated infected erythrocytes incubated with NBD-Sph for 1 h and (ii) determination of NBD-S1P level in isolated host free parasites directly incubated with NBD-Sph, followed by DMS treatment. The results clearly indicated that, when host free parasites isolated from the infected erythrocytes with 1 h prior incubation with NBD-Sph, the NBD-S1P level was found to be significantly high, which was then predominantly reduced to ~ 50% following treatment with DMS suggesting parasite co-dependency to host S1P pool (Fig. 1C (ii)). On the contrary, the isolated host free parasite didn’t shown any change in NBD-S1P level in presence of DMS suggesting absence of parasite-specific S1P synthesis machinery (Fig. 1C (ii)). Representative scheme for these results depicted that parasite inside the host strictly codependent on host S1P, and it does have its own S1P synthesis machinery (Fig. 1A).

**Figure 2:**
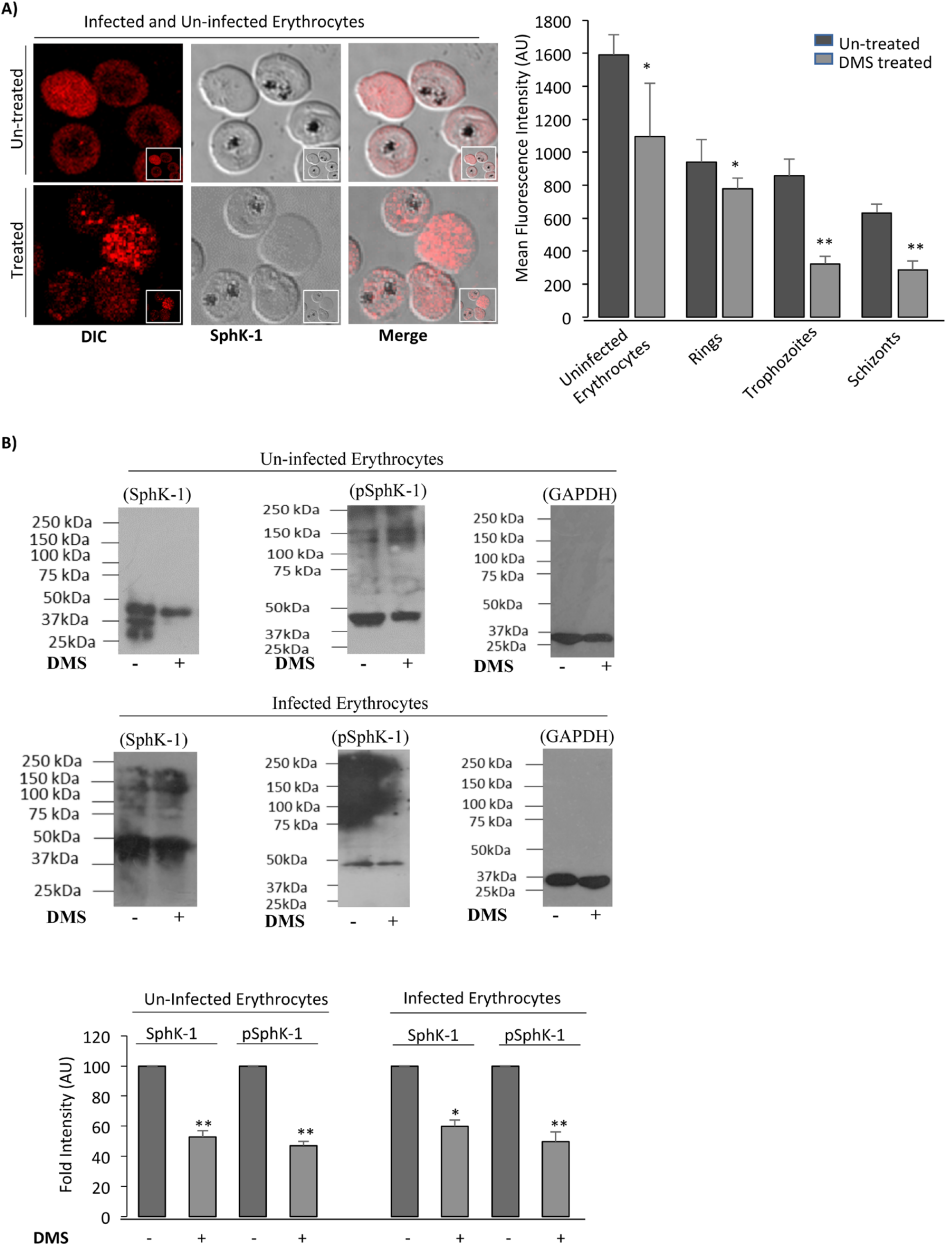
Inhibition of host SphK-1 by specific inhibitor DMS causes decrease in SphK-1 protein level and phosphorylation. (**A**) Confocal micrographs demonstrate altered host SphK-1 level in mixed-stage parasite culture following treatment with DMS (14 µM). Bar graph denotes the differential mean fluorescence intensity (MFI) denoting host SphK-1 level in infected and uninfected erythrocytes following DMS treatment. The supportive immunofluorescence micrograph was incorporated in Supplementary Fig. 4. (**B**) The representative uncropped immunoblots show phosphorylation status of SphK-1 and its level in treated and untreated cells. Total cell lysates probed with GAPDH was used as a loading control. The graph represents fold change in the band intensity in individual lanes for two individual experiments. Three independent experiment have been performed, n = 3, ∗*p* ≤ 0.05.

Physiologically, the main reservoir of circulating S1P is known to be erythrocytes that are rich in SphK-1 while devoid of S1P lyase and sphingolipid transporter 2 (SPNS2)^38,39^. Hence, modulation of SphK-1 level could mediate change in the levels of circulating or intracellular S1P concentrations. Therefore, we determined the level of SphK-1 in parasite infected and uninfected erythrocytes by immunolabelling and immunoblotting. For immunolabelling, mixed stage parasites were probed with anti-SphK-1 antibody. Significant reduction in SphK-1 levels was observed following treatment with DMS in un-infected and infected erythrocytes as compared to untreated control (Fig. 2A). The images were included in (Supplementary Fig. 3).

To further understand, if host Sphk-1 activity might be governed by phosphorylation, we have evaluated the phosphorylation status of host SphK-1 in presence of its specific inhibitor (DMS), by probing lysate of infected erythrocytes with specific Phospho-SphK-1 (Ser225) antibody with GAPDH used as a loading control. In line with the previous result of immunolabelling, prominent reduction in both SphK-1 protein and its phosphorylated form (pSphK-1) were detected in infected and uninfected erythrocytes following DMS treatment as compared to the untreated control (Fig. 2B). The uncropped blots pictures were included in (Supplementary Fig. 4). DMS block the activities of SphK1 by competing with the natural substrate sphingosine. This finding was found to be in sync with a recent study, which showed that SphK-1 inhibitors could induce ubiquitin-proteasomal degradation of SphK-1 in solid cancer cell lines^40,41^ and proliferation of vascular smooth muscle cells^42–44^. The ubiquitin-proteasomal degradation of SphK-1 is associated with binding and/or catalytic activity inhibition of SphK-1. However, other SphK-1 and SphK-2 inhibitors, such as SKi (2-(p-hydroxyanilino)-4-(p-chlorophenyl) thiazole) induce proteasomal degradation of SphK1 through a mechanism only partially dependent on direct binding to SphK-1^43^. Overall these results indicated a decreased level of S1P and Sphk-1 activity that could be the reason underlying downregulation of SphK-1 protein after treatment with DMS.

### Inhibition of erythrocytic SphK-1 triggers parasite progression and death

**Figure 3:**
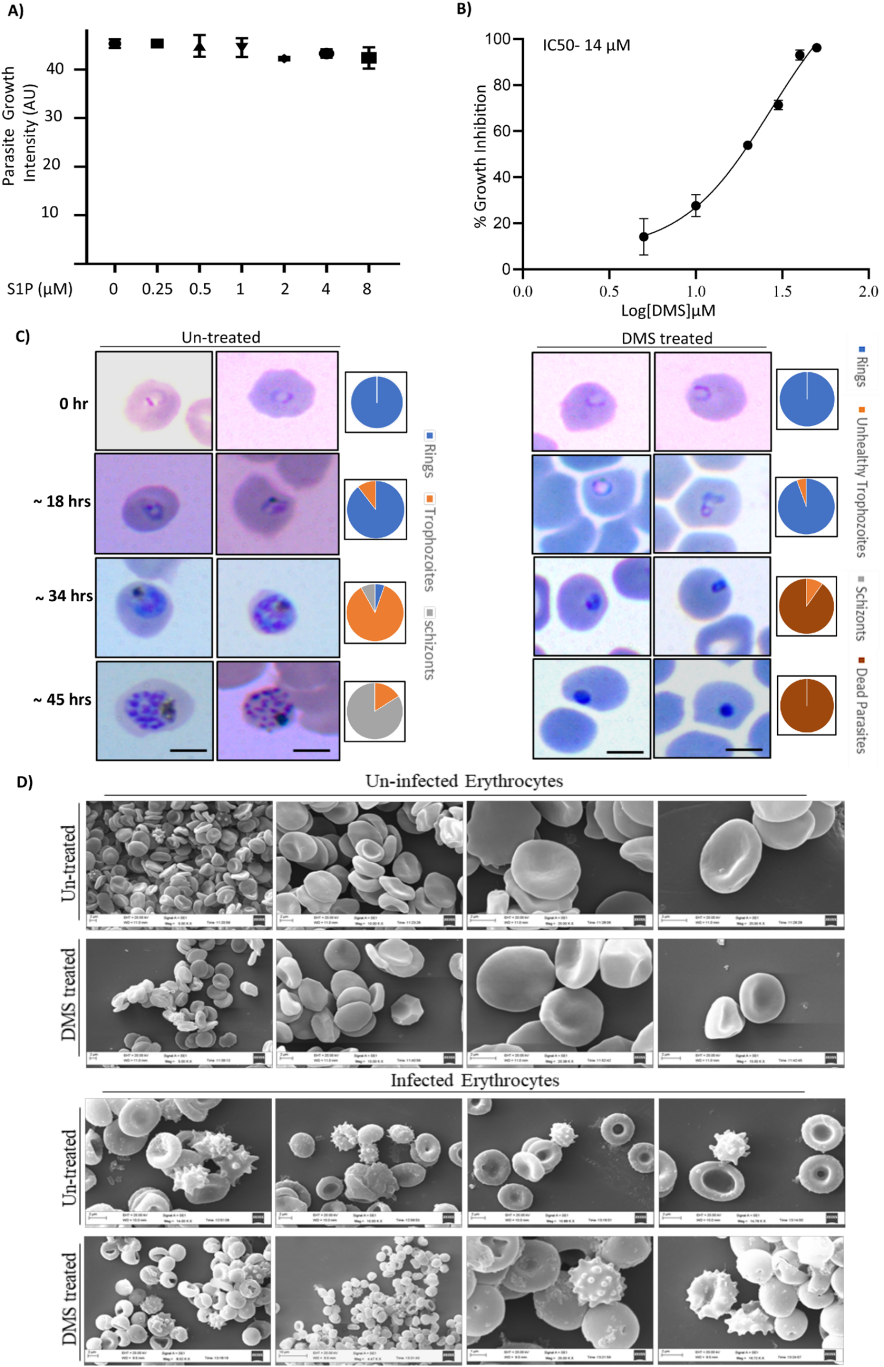
Inhibition of host SphK-1 blocks parasite growth and progression. (**A**) Parasite-infected erythrocytes mixed with erythrocytes to a final parasitemia of 1% and final hematocrit of 2% and incubated with S1P at different concentration (0–8 µM) for 48 h. (**B**) Percentage inhibition of *P. falciparum* growth was evaluated at different DMS concentrations (0-40 μM), as presented in the bar graph. Three independent experiments were performed in triplicates with 96-well plates using a SYBR-green assay. The IC50 value was determined as 14 µM for the parasite growth inhibition with DMS. (**C**) Visualization of stage specific inhibition of *P. falciparum* progression following DMS treatment was depicted by light microscopic images of Giemsa stained ring, trophozoite and schizont stages. (**D**) Scanning electron micrographs of DMS-treated infected and uninfected erythrocytes represented comparative morphometric analysis. Scale bars = 5 μm. Three biological replicates have been performed n = 3, ∗*p* ≤ 0.05.

To decipher the link between host SphK1 activity and parasite growth, we have elucidated the effects of DMS and S1P on synchronized parasite cultures at ring stage. DMS treatment was done in an increasing manner (0, 5, 10, 20, 30 and 40 µM) and S1P treatment was performed at various concentrations (0, 0.25, 0.5, 1, 2, 4 and 8 µM) for a time period of 48 h separately. After completion of one asexual life cycle of the parasite, SYBR-green was added for assessing the percentage inhibition by S1P and DMS. The data revealed a moderate to potent anti-malarial activity of DMS with a significant reduction in the parasite load, with IC_50_ value attended at 14 µM. This result clarified that there is no impact of extracellular S1P on parasite growth. Conversely, blocking the intracellular S1P synthesis in host by DMS showed detrimental effect on parasite growth (Fig. 3A,B). To examine its impact on stage specific inhibition of parasite progression, highly synchronized ring stage parasites were treated with 14 µM DMS and their development were monitored at different time intervals (0, 18, 34, 45 hpi) by preparing thin blood smears of the treated and untreated infected erythrocytes. As observed in the blood smears, DMS drastically affected progression of the parasite from rings to trophozoites, stalling the parasite progress at the ring stage as compared to the untreated parasite infected control. Halt in the parasite growth could be accounted to the formation of ‘pyknotic body’ after 36 h of the treatment (Fig. 3C). This progression arrest coincided with a significant drop in the S1P levels of the erythrocytes after DMS treatment. These findings strongly support the hypothesis, that treatment with DMS can lead to impaired de-novo synthesis of S1P in host erythrocytes. Thus, in case of infected erythrocytes, parasite were no longer able to utilize the S1P pool from the host, ultimately resulting in attenuated growth pattern (Fig. 3C).

Inhibition of SphK-1 causes less production of S1P, which plays an important role in *Plasmodium* growth and development in the erythrocytes. Further, we have ascertained whether the host SphK-1 inhibition mediated retardation of parasite growth and progression is independent of DMS-induced topological aberration in host membrane. To answer this, we have performed SEM-based imaging of both infected and uninfected erythrocytes in presence of DMS. The SEM micrographs does not show any significant changes in infected erythrocytes, following DMS treatment as compared to the respective untreated controls (Fig. 3D). Similar observation could also be inferred from the experiments involving uninfected and infected erythrocytes treated with DMS (Fig. 3C).

### Inhibition of erythrocyte SphK-1 resulted in reduced glucose uptake and lactate production

**Figure 4:**
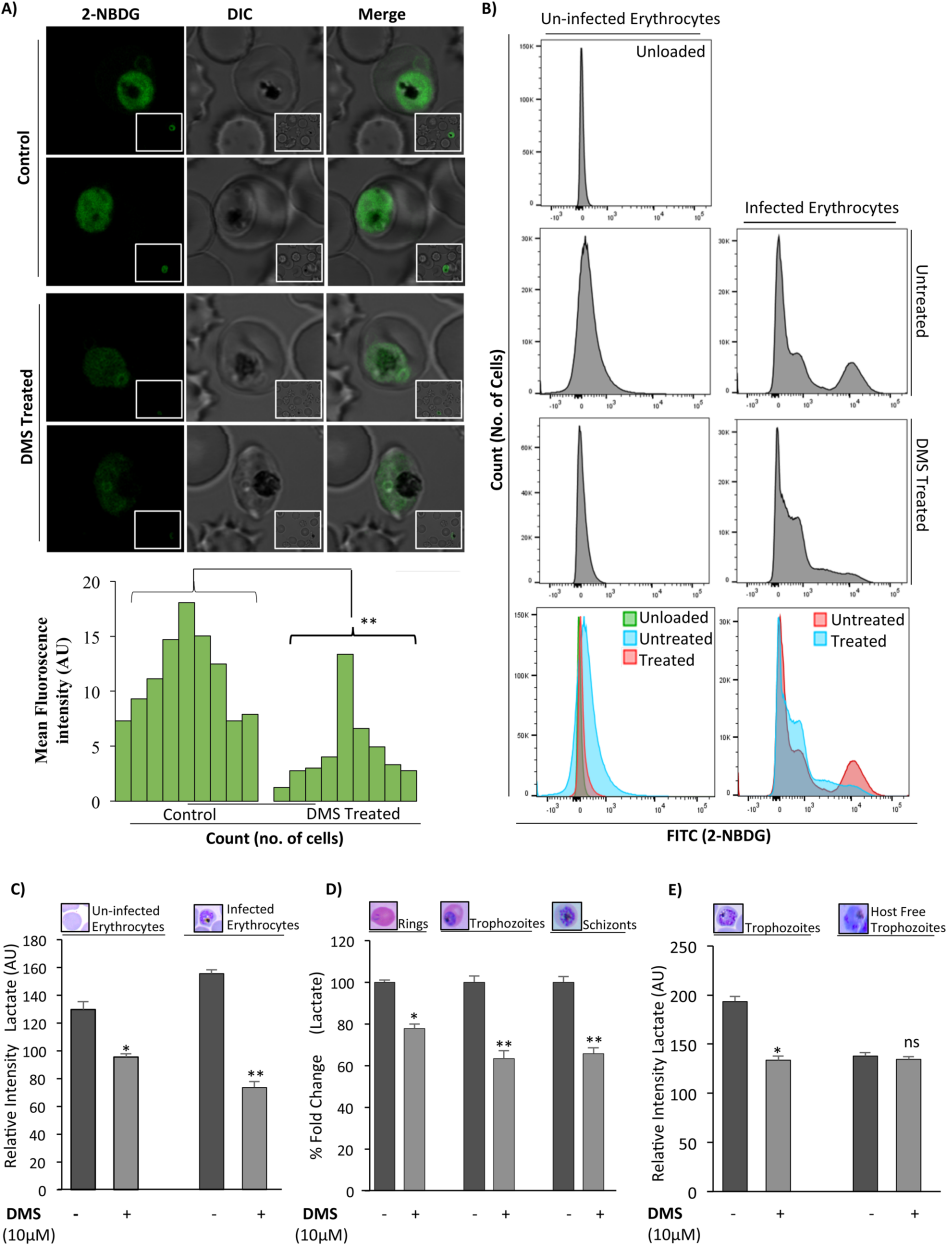
Inhibition of host SphK-1 results in reduced glucose uptake and lactate production. (**A**) Evaluation of 2-NBDG uptake in parasite infected erythrocytes following DMS treatment by live-cell imaging. Respective MFI of individual infected erythrocytes were plotted against individual untreated cells. Three independent experiments have been done, n = 3, ∗∗*p* ≤ 0.01. The representative live cell images were included in Supplementary Fig. 5 (**B**) Representative histograms depict changes in number of FITC^positive^ population in flow cytometry analysis correlating to 2-NBDG uptake following DMS treatment in infected erythrocytes. (**C**) Quantification of lactate in uninfected and infected erythrocytes treated with DMS (14 μM), was represented as changes in relative intensities. (**D**) Evaluation of lactate levels in asexual blood stages following DMS treatment, was plotted as percentage fold change. (**E**) Trophozoites and saponized/host-free trophozoites were used for experiment. Representative bar graph displayed changes in lactate levels as relative intensities for trophozoites and saponized/host-free parasites. The graph represents data for two individual experiments ∗*p* ≤ 0.05.

Previous study has shown that the intracellular S1P in erythrocytes has been linked to regulation of glycolysis^45^. Since erythrocytes are solely dependent on glycolytic pathway to meet their energy requirements, we asked whether blocking S1P production in erythrocytes has any impact on the intraerythrocytic growth of parasites. Towards this, we measured the glucose uptake in parasite-infected erythrocytes via fluorescence imaging and fluorescence-activated cell sorting (FACS) using a non-metabolizable glucose analog, 2-NBDG, which is fluorescently tagged. 2-NBDG accumulates inside the cells by entering through the glucose transporters but it does not enter into the glycolysis pathway^46,47^. Parasite infected erythrocytes were pre-incubated with DMS or solvent control for 3 h at 37 °C. The cells were then incubated with fluorescently labeled 2-NBDG and its uptake was measured after washing the cells with PBS using confocal microscopy. Live-cell imaging of DMS-treated infected erythrocytes represented drastic reduction in glucose uptake, as represented by diminished green fluorescence indicating lower uptake of 2-NBDG, while, the untreated infected erythrocytes displayed high uptake of glucose analogues (Fig. 4A). the un zoomed images included in (Supplementary Fig. 5). The fluorescence intensities were plotted by measuring ten individual cells using Image J for the treated and untreated cells indicating the same (Fig. 4A). This finding confirmed that impaired uptake of glucose analogue in DMS-treated infected erythrocytes was due to dysregulated glycolysis but not compromised cell viability, using Syto9/Propidium Iodide assay (BacLight; Molecular Probes, Eugene, OR, USA) (data not shown). Further, flow cytometry analysis of 2-NBDG uptake in infected erythrocytes demonstrated a 10-fold shift in fluorescence intensity indicating enhanced uptake of 2-NBDG, whereas, the DMS-treated infected erythrocytes showed severe depletion in its uptake, as shown by diminished intensity peak in representative histograms (Fig. 4B).

Further to have an in-depth understanding of SphK-1 mediated glycolytic pathway modulation in infected erythrocytes, we investigated the impact of DMS treatment on total lactate production in infected and uninfected erythrocytes. The intra-erythrocytic stages of *P. falciparum* lack a functional citric acid cycle and is largely reliant on glycolysis to fulfill its very substantial energy requirements^6,48^. In addition, erythrocytes infected with mature trophozoite stage of parasites consume glucose up to two folds of magnitude faster than uninfected erythrocytes, eventually converting it to lactate^23,24,46^. Thus, to dissect this mechanism of SphK-1 dependent growth progression in parasite infected erythrocytes, and its interlink to glycolysis, we estimated the amount of lactate production in both infected and uninfected erythrocytes in presence of DMS for 3 h. The findings suggested a significant reduction in lactate generation in DMS-treated infected and uninfected erythrocytes, as compared to respective untreated controls (Fig. 4C). Interestingly, parasite stage-specific estimation of lactate levels during intra-erythrocytic cycle, represented predominant decrease in lactate generation in presence of DMS, as clearly evident in rings, trophozoites and schizonts (Fig. 4D). These data depicted that; lowering of S1P levels by DMS-based inhibition of host SphK1 can lead to compromised glycolysis, which is then manifested as reduced lactate levels in intra-erythrocytic cycle of *P. falciparum*.

To gain further insight, whether DMS can impose direct impact on parasites or indirectly affect through host-mediated altered glycolysis, we have elucidated the lactate levels both in the trophozoite stage of parasite and in saponin lysed host-free parasites following DMS treatment for two hours. For saponin lysis the maintained trophozoite culture were lysed according to protocol reported previously^49^. Interestingly, the lactate levels were found to be significantly lower as compared to their respective untreated controls in DMS treated trophs, except in case of saponin lysed parasites wherein, no significant difference in the lactate levels could be detected (Fig. 4E). Reduced levels of lactate production could be accountable either to the down-regulated glycolysis rate and/or reduced glucose uptake in trophozoite infected and uninfected erythrocytes. However, DMS treatment of saponin lysed host-free trophozoites demonstrated negligible difference in the lactate levels as compared to the untreated control (Fig. 4E). Altogether, these readouts suggested that host SphK-1 contributes to the regulation of glycolysis and its effect on parasite growth is mediated solely via the host erythrocytes.

### Abrogated S1P synthesis in infected erythrocytes led to impaired translocation of glycolytic enzyme, GAPDH

**Figure 5:**
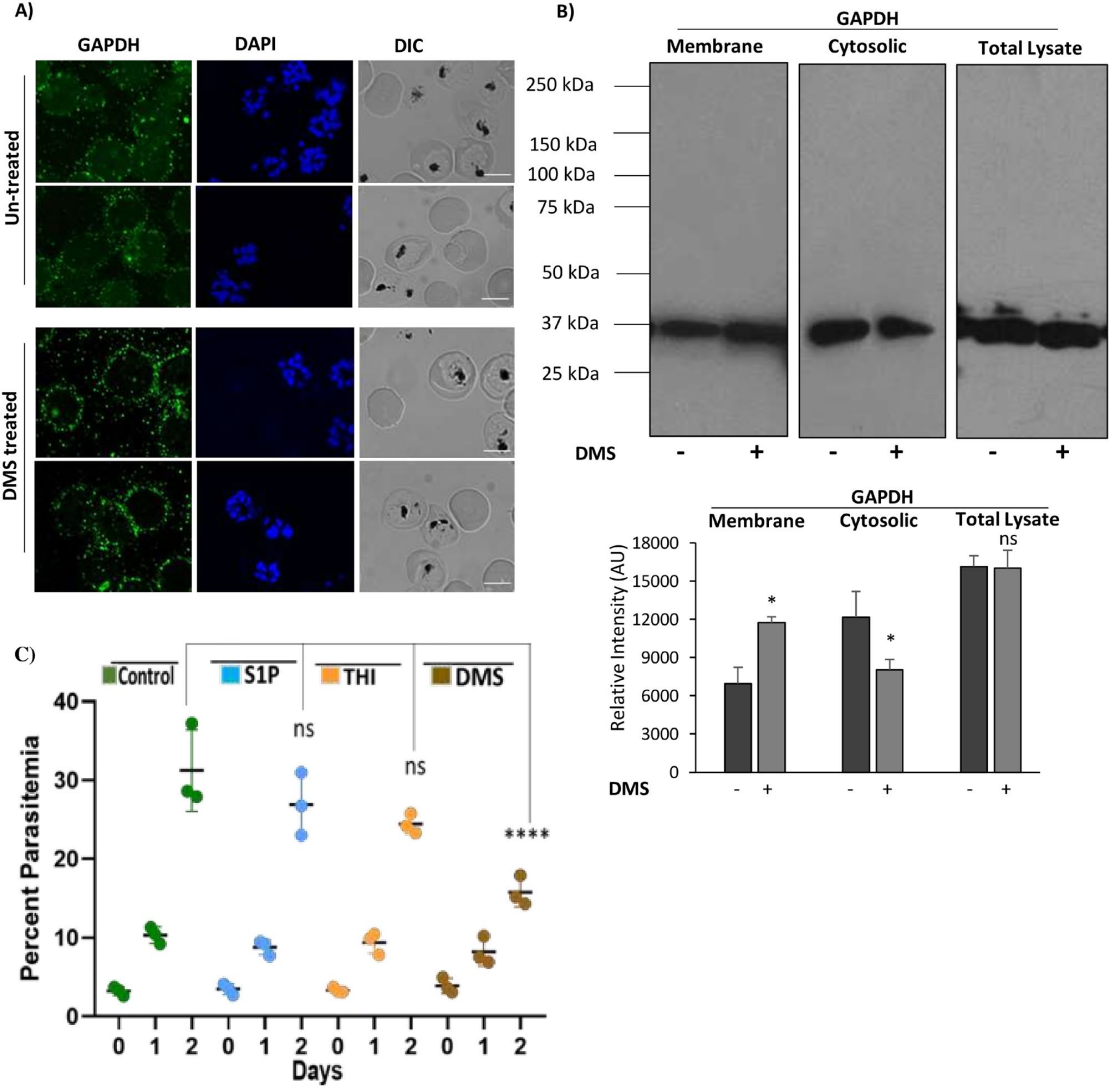
Translocation of GAPDH in parasite infected erythrocytes upon host SphK-1 inhibition. (**A**) Confocal micrographs demonstrate GAPDH level and localization in both DMS-treated and untreated infected erythrocytes. (**B**) Detection of GAPDH in both membrane and cytosolic fractions of DMS-treated infected erythrocytes. Total cell lysate was used as a loading control. Change in the level was plotted as relative intensities of bands detected in different cellular fractions. Three independent experiments have been done, n = 3, ∗ p ≤ 0.05. (**C**) Antimalarial effects of S1P, THI, DMS. Groups of BALB/c mice (n = 3 per group) were inoculated with 1 × 10^8^ parasitized erythrocytes of pbANKA by IP injection. Post 48 h of infection; DMS was administered by intraperitoneal route at 4 µg/kg; oral administration of 2-Acetyl-4-tetrahydroxybutyl imidazole (THI) was done at 4 µg/kg and S1P was injected intravenously at a concentration of 0.4ug/kg. Untreated control mice received 10 ml/kg of DW. The treatment was carried out for 3 consecutive days (days 0–3). Every day parasitemia was determined by microscopic examination of Giemsa stained thin blood smears. Comparative analysis among the groups, namely; DMS, THI and S1P treated mice with untreated mice done with One-way ANOVA followed by Tukey multiple comparison test, which revealed significant differences between DMS treated and untreated mice with p values, *p* < 0.0001****, not significant (ns).

Since, host SphK-1 inhibition led to depletion in S1P levels in infected erythrocytes, we assumed that, the translocation of GAPDH from membrane to cytosol might be also hampered in DMS-treated infected erythrocytes. To validate the same, we checked the levels of GAPDH in cytosolic and membrane fractions of parasite infected and uninfected erythrocytes, by immunoblotting and immunolabelling. The confocal imaging revealed that, following DMS treatment GAPDH was mainly restricted to the erythrocytic membrane rather than translocating to the cytosol, suggesting decreased glycolysis (Fig. 5A). The total cell lysate was used as a control. After DMS treatment, both the membrane and cytosolic fractions were separated for probing with anti-GAPDH mouse antibody. As expected, DMS treatment reduced the translocation of GAPDH from the erythrocyte membrane to cytosol, as evident in immunoblot and its respective band intensities in both membrane and cytosolic fractions (Fig. 5B). The uncropped blots picture were included in (Supplementary Fig. 6). These findings confirmed reduced release of GAPDH to the cytosol due to altered glycolysis in DMS-treated infected erythrocytes that strongly supports the results obtained from immunofluorescence. Collectively, these results suggested diminished S1P levels in DMS-treated infected erythrocytes can lead to reduced glycolysis.

### In vivo antimalarial activities

**Figure 6:**
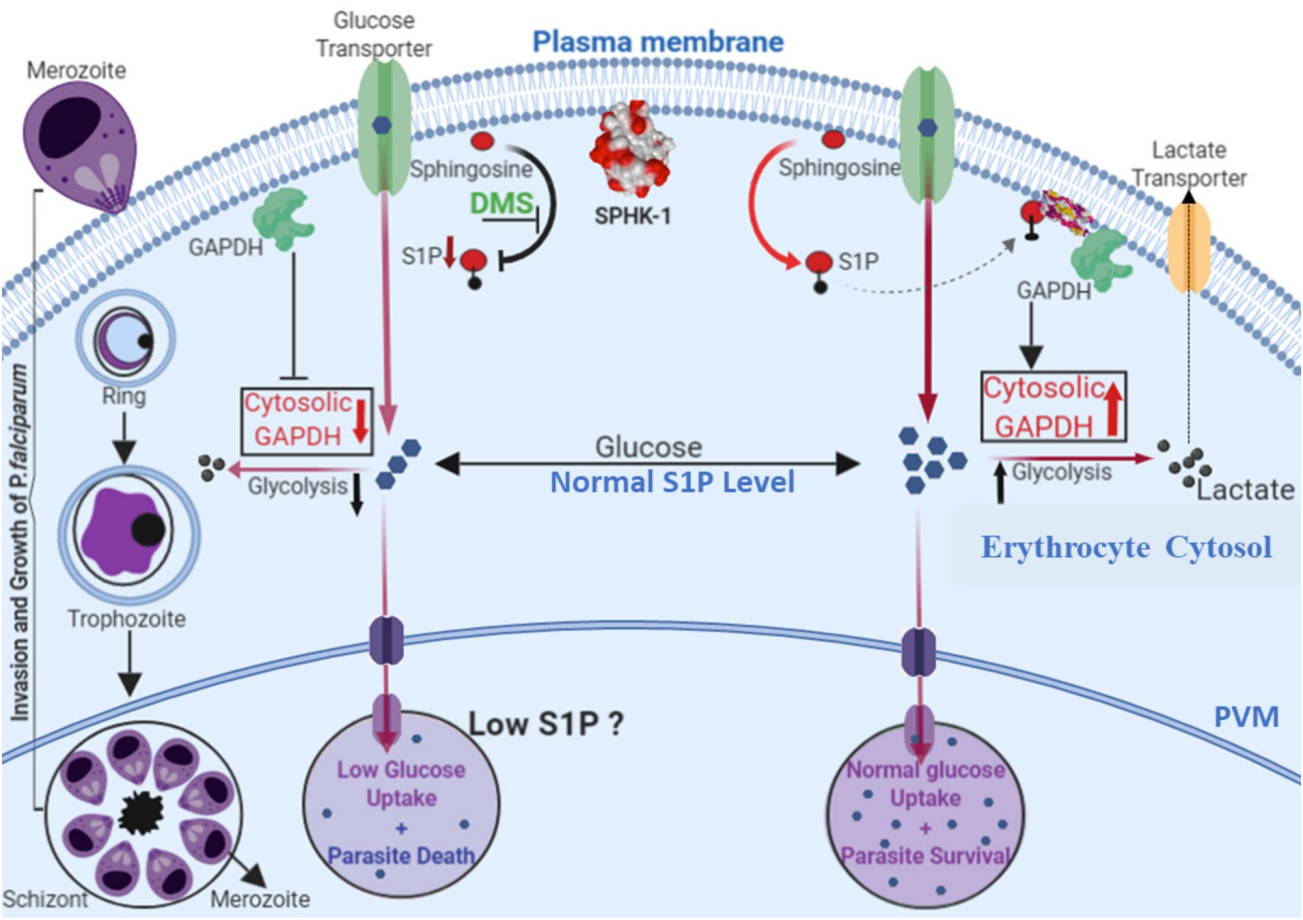
In the proposed working model, life cycle of blood stage parasite is shown. In normal condition, S1P bind to deoxy-Hb and facilitates binding of deoxy-Hb to membrane and release of GAPDH; increased cytosolic GAPDH accelerates glycolysis and generate lactate which is a by-product of glycolysis and does not stall the growth of parasite. In case of DMS-mediated inhibition of host SphK-1, S1P level gets reduced, leading to altered binding with deoxy-Hb. Thus, it does not facilitate GAPDH to cytosol due to which glycolysis is suppressed leading to retarded parasite growth.

Animals receiving DMS, exerted potent antimalarial activity against PbA at dose 4 µg/kg (Fig. 5C). In parallel antimalarial effects of both S1P and THI were evaluated in animals infected with PbA. The significant inhibition of parasitemia enforced by the DMS was observed at 4 µg/kg. Moreover, the mice treated with THI and S1P did not show statistically significant difference as compared to the DMS-treated group. These data demonstrated that DMS inhibits PbA but needs to be administered at high dose and for prolonged time to eradicate PbA infection in mice. The model shows that host SphK-1 inhibition can abolish intra-erythrocytic growth and progression of *P. falciparum* and secondly, reduced S1P levels can lead to the altered host glycolysis, causing parasite growth retardation (Fig. 6).

## Discussion

In malaria, *Plasmodium falciparum* is known to employ lipids from its de novo pool and host cell lipid repertoire^50^ during its growth and proliferation in ABS, suggesting a defined crosstalk of metabolic circuits that can be exploitable for designing antimalarials targeting the metabolic co-dependency of parasite. Metabolic profiling of infected erythrocytes revealed significant alterations in various host-lipid metabolites, including sphingolipids, the unique class of structural biolipids. Erythrocyte is the major reservoir for circulating S1P as compared to other peripheral tissues, due to lack of S1P degrading enzymes (S1P lyase and S1P phosphohydrolase), and its capability to import sphingosine from extracellular environment^38,39^. It is noteworthy that, S1P and its concentration gradient from circulating to peripheral tissues governs several critical physiological processes, including vascular integrity, trafficking of lymphocytes and bone homeostasis etc.^51–53^. Accumulating body of evidences revealed strong role of deregulated S1P metabolism in malaria patient infected with *P. vivax* and *P. falciparum* and correlated with thrombocytopenia and anemia^54^, suggesting that S1P signaling cascade might be implicated in the severity of malaria^14^. Since *Plasmodium* do not harbor any form of SphK-1 to synthesize S1P from its precursor Sph de novo, we hypothesized that *P. falciparum* might modulate host-SphK1 activity for its growth and progression during intracellular development. To prove this hypothesis, we used SphK-1-specific inhibitor, DMS to evaluate the S1P production in infected erythrocytes, using LC/MS-MS, ELISA and metabolic labelling (Fig. 1). Our findings suggested that blocking of host-SphK-1 in infected erythrocytes promptly diminished the level of S1P in both infected and uninfected erythrocytes, as evident by reduced uptake of NBD-S1P, a metabolic probe for host-S1P in both infected and uninfected erythrocytes. Since parasites do not have any form of SphK-1, we then asked if blocking host-SphK-1 by DMS has any direct impact on S1P levels in host-free parasites. The finding suggested that NBD-S1P uptake in host-free parasite was significantly low as in case of DMS treatment as compared to untreated control (Fig. 1).

Further to authenticate that, if the parasites are strictly acquiring host-S1P, we used saponin lysed host-free parasites and incubated further with DMS. As assumed, parasites did not uptake NBD-S1P, suggesting its inaccessibility to host-specific NBD-S1P in absence of host (Fig. 1C (ii)). We then evaluated if DMS treatment would have any impact on SphK-1 and its phosphorylation levels in ABS and uninfected erythrocytes. The results suggested DMS treatment could drastically reduce the both SphK-1 level and the phosphorylation status in both infected erythrocytes and uninfected controls (Fig. 2). Since parasite uptakes the S1P from intracellular level during ABS, we then evaluated if exogenous addition of S1P to infected erythrocytes has any impact on its growth and progression. The results clearly indicated that extracellular supplementation of S1P had no impact on parasite growth and progression, however treatment with DMS demonstrated profound growth inhibition with formation of prominent ‘pyknotic bodies’ at IC50 (14 μM) (Fig. 3A–C), suggesting that the parasites have only access to intracellular pool for their growth in ABS. This finding suggested that, since erythrocytes mainly harbor SphK-1 with no S1P receptor for its innate signaling, thus inhibiting the host-SphK-1 by DMS might attenuate parasite’s co-dependency on host-S1P metabolism for its survival. Next, to negate the possibility of DMS-enforced topological alterations in infected erythrocytes as the basis of aborted parasite growth and invasion, we have performed SEM-based analysis of both uninfected and infected erythrocyte membranes after DMS treatment. To highlight, there was no significant changes could be identified in membrane structures of infected erythrocytes even after DMS treatment (Fig. 3D). Similar inference was drawn from the experiments involving uninfected erythrocytes with DMS (Fig. 3D).

Previous reports have shown that during *Plasmodium* infection, erythrocytes demonstrated 6-fold increase in phospholipids (PL), along with a sharp rise in glycolytic flux, with glucose uptake up to 50 folds, predominant in metabolically progressive stages such as (trophozoite and schizont)^7,55^. suggesting host-dependency of the parasite for fulfilling its metabolic needs^28^. Interestingly, S1P triggers glycolysis in erythrocytes in response to hypoxic stimuli and subsequent release of GAPDH, the enzyme involved in host glycolysis^26^. Since parasite lacks de novo pathways for both S1P metabolism and ATP generation from TCA cycle, we have tried to decipher if blocking the host-SphK-1 activity might modulate the glycolysis during metabolically active stages of *P. falciparum*. To explore this, we have also elucidated the impact of host SphK-1 inhibition on glucose uptake, during intra-erythrocytic development by measuring the live uptake of glucose using a fluorescent-labeled glucose analogue (2-NBDG). The results strongly indicated diminished glucose uptake in DMS-treated infected erythrocytes, imposing a direct role of host-SphK-1 in regulation of host-glycolysis (Fig. 4). In addition, we have taken trophozoites and schizonts as the metabolically active forms of intracellular developmental stage of *P. falciparum* and estimated the lactate generation in the presence of DMS. The results depicted, drastic switch of metabolically active stage of parasites to metabolically dormant state, leading to parasite growth retardation (Fig. 4). This data strongly advocated the role of host-SphK1 in regulation of glycolysis-dependent growth and progression of parasites. Further to ascertain, if the altered glycolysis of intra-erythrocytic cycles is mediated specifically through erythrocytes not via the parasites; we also measured the lactate levels in DMS-treated saponized/host-free parasites, as a proof-of-concept. The result clearly ruled out the involvement of parasite-mediated glycolysis, as no change in lactate levels could be detected in host-free parasites following DMS treatment (Fig. 4). According to a recent study by K Sun et al., SphK-1 activity gets elevated in erythrocytes under hypoxic conditions, leading to enhanced S1P level and thereby, increasing its binding to deoxygenated hemoglobin (deoxy-Hb). Subsequently, this facilitates deoxy-Hb anchorage to the membrane, leading to enhanced translocation of membrane-bound GAPDH to the cytosol, resulting in increased glycolysis^45^. To further evaluate, whether abolishing the host-SphK-1 activity in DMS-treated infected erythrocytes would also dysregulate the GAPDH level and destabilize its translocation, we have elucidated the level of GAPDH both in membrane and cytosolic fractions. The findings unravelled reduced translocation of GAPDH from membrane-to-cytosol in DMS-treated infected erythrocytes, as evident from altered localized level of GAPDH, in both immunoblot and confocal micrographs (Fig. 5). Further to authenticate the in vitro findings in vivo*,* we have used PbA mice models and treated with DMS, S1P and THI separately, to elucidate the impact of host-SphK-1 and S1P levels on parasite growth. Mice treated with DMS showed significant reduction in parasitemia as compared to untreated control, whereas mice treated with THI and S1P demonstrated no significant reduction in parasitemia, suggesting hardly any impact of increased plasma S1P level on growth of parasites. This data strongly indicated parasite co-dependency on host intracellular pool of S1P (Fig. 5).

To summarize, our study highlights two important findings; firstly, the inhibition of host SphK-1 activity can abrogate the intra-erythrocytic growth and progression of *P. falciparum,* and secondly, diminished S1P levels can lead to host-mediated altered glycolysis resulting in growth retardation of parasites (Fig. 6). Overall, this study introduces SphK-1/S1P signaling nexus in erythrocytes as the alternate pathway for *P. falciparum* survival. Since, knocking down of host SphK-1 is not lethal^56^, thus targeting the same might eliminate the problem of resistance, aiding in developing novel anti-malarial chemotherapeutics.

## Supplementary Information

Supplementary Figures.
