# Supplementary material for: Erythrocyte sphingosine kinase regulates intraerythrocytic development of Plasmodium falciparum
Source: Sci Rep. 2021 Jan 13;11:1257. doi: 10.1038/s41598-020-80658-7 (PMC7806667; doi:10.1038/s41598-020-80658-7)
Supplement: Supplementary file 1 — Supplementary Figures. [file 41598_2020_80658_MOESM1_ESM.pdf]

Erythrocyte Sphingosine Kinase Regulates Intraerythrocytic Development of *Plasmodium falciparum*

Raj Kumar Sah[1#], Soumya Pati[2#], Monika Saini[1-2] and Shailja Singh[1*]

[1]Special Centre for Molecular Medicine, Jawaharlal Nehru University, New Delhi, India
[2]Department of Life Sciences, School of Natural Sciences, Shiv Nadar University, Greater Noida, India

***Correspondence:**
Shailja Singh
shailja.jnu@gmail.com
shailja.singh@jnu.ac.in

Key words: Sphingosine, Sphingosine-1-phosphate, Sphingosine kinase Erythrocyte, plasmodium, malaria

A)

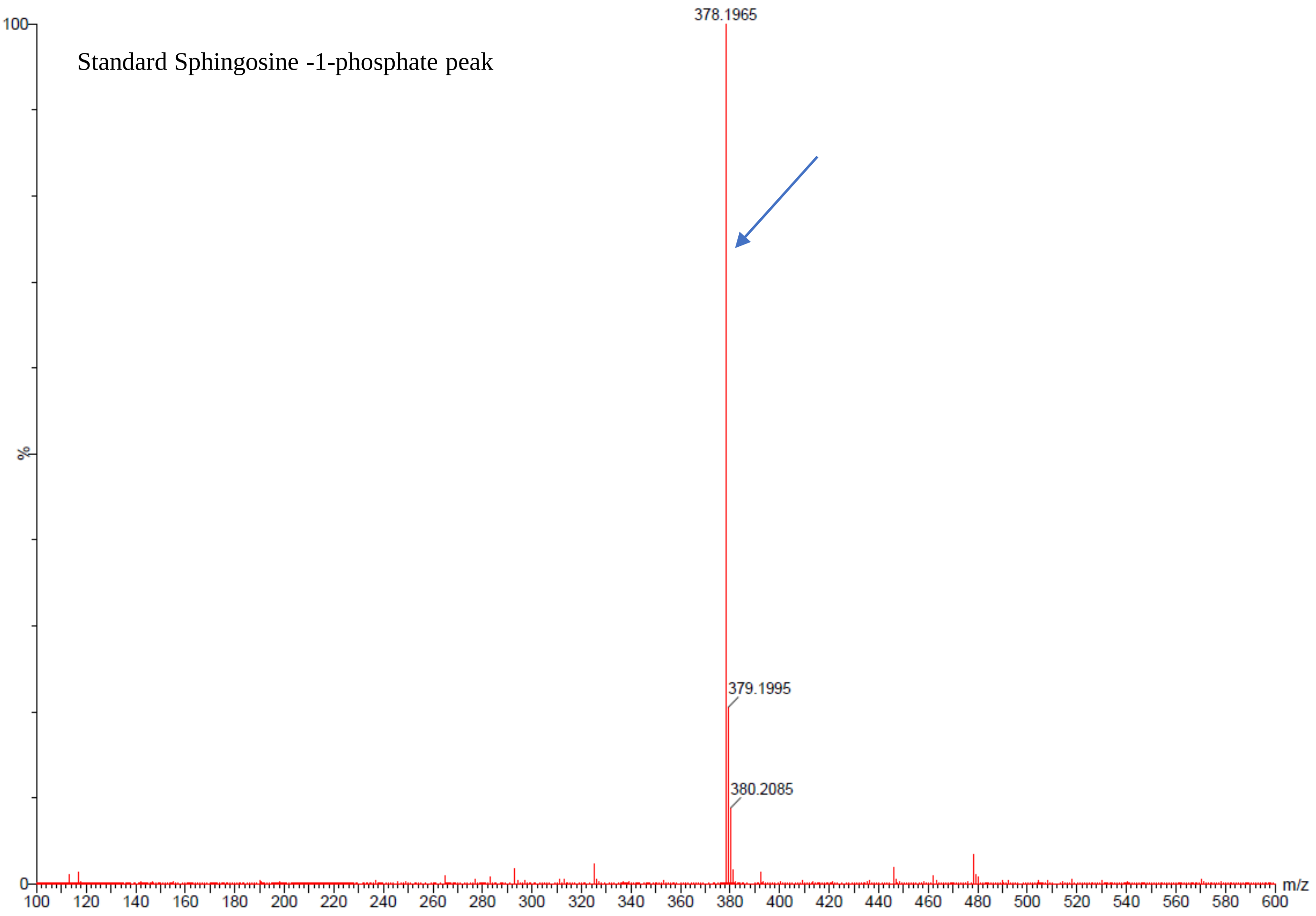

B)

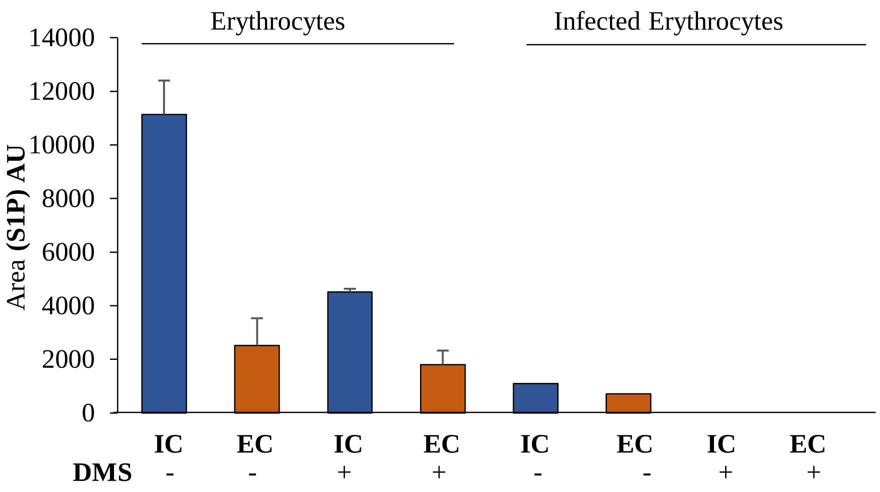

Supplementary Figure 1. Characteristic peak of S1P in MS spectra. S1P in methanol was subjected to LC/MS analysis and generated a characteristic peak at position 378.16 acquired in MS spectra **(A)**. Representative bar graphs display LC/MS-based quantification of intracellular (IC) and extracellular (EC) S1P levels in parasite infected and uninfected erythrocytes in presence of DMS (14 µM). Acquired S1P-specific peak highlighted in the MS spectra **(B)**.

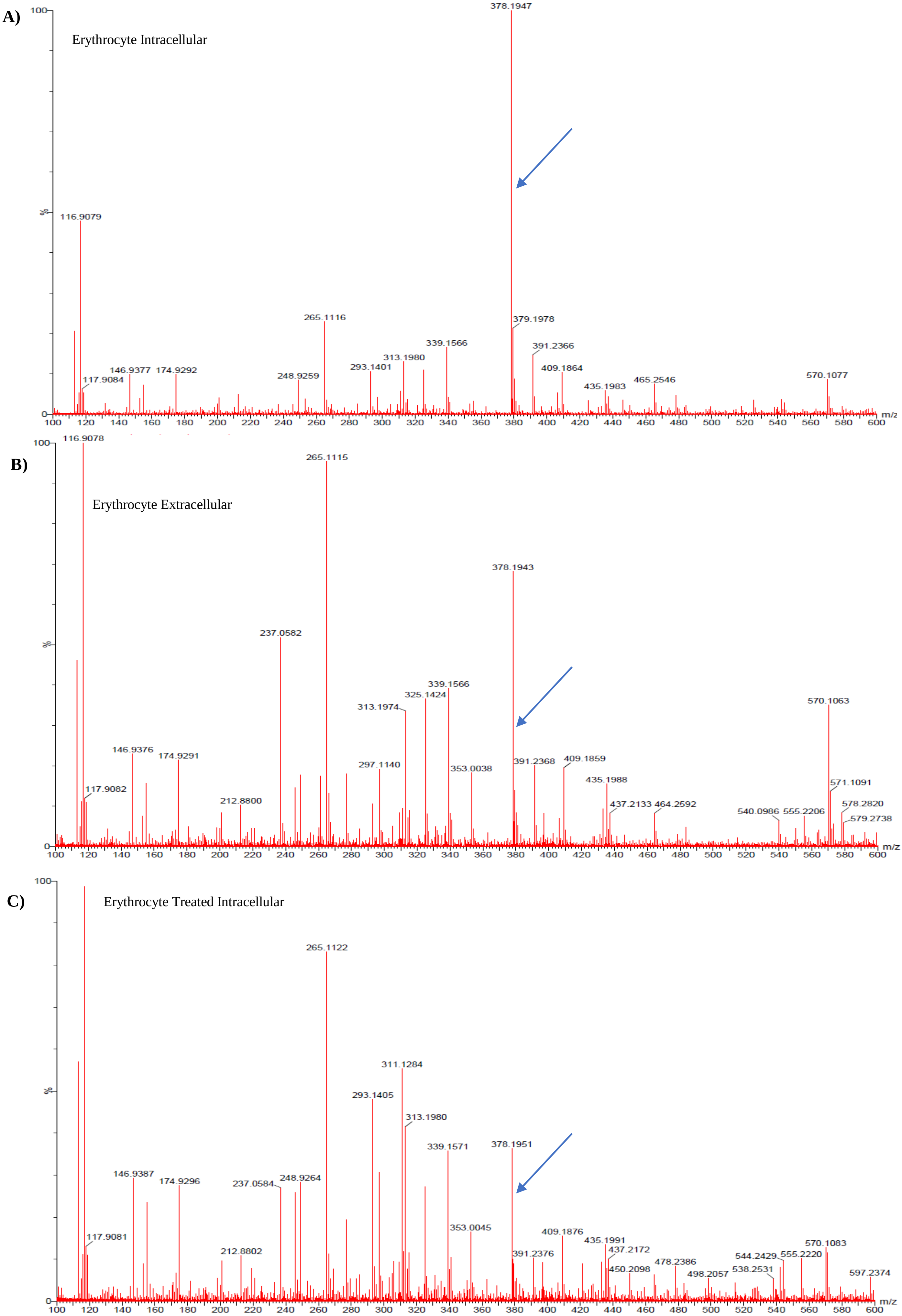
A)
Erythrocyte Intracellular
378.1947
116.9079
265.1116
379.1978
339.1566
391.2366
313.1980
293.1401
409.1864
146.9377
174.9292
248.9259
117.9084
465.2546
435.1983
570.1077
B)
Erythrocyte Extracellular
116.9078
265.1115
378.1943
237.0582
339.1566
325.1424
313.1974
570.1063
146.9376
174.9291
297.1140
353.0038
391.2368
409.1859
117.9082
212.8800
435.1988
571.1091
437.2133
464.2592
540.0986
555.2206
578.2820
579.2738
C)
Erythrocyte Treated Intracellular
265.1122
311.1284
293.1405
313.1980
339.1571
378.1951
146.9387
174.9296
237.0584
248.9264
353.0045
409.1876
117.9081
212.8802
435.1991
437.2172
391.2376
570.1083
544.2429
555.2220
478.2386
450.2098
538.2531
498.2057
597.2374
%
m/z

D)

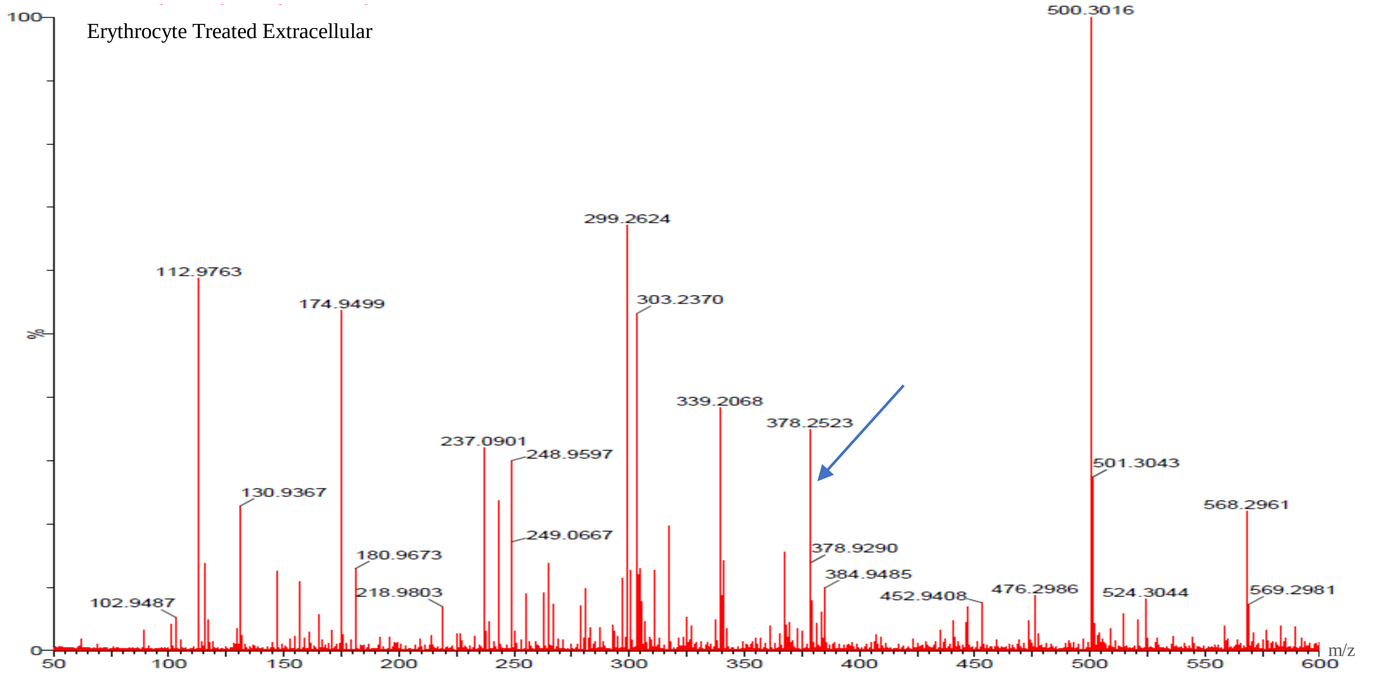
Erythrocyte Treated Extracellular
100
%
0
500.3016
299.2624
112.9763
174.9499
303.2370
339.2068
378.2523
237.0901
248.9597
501.3043
130.9367
568.2961
249.0667
180.9673
378.9290
218.9803
384.9485
102.9487
476.2986
452.9408
524.3044
569.2981
m/z
50
100
150
200
250
300
350
400
450
500
550
600

E)

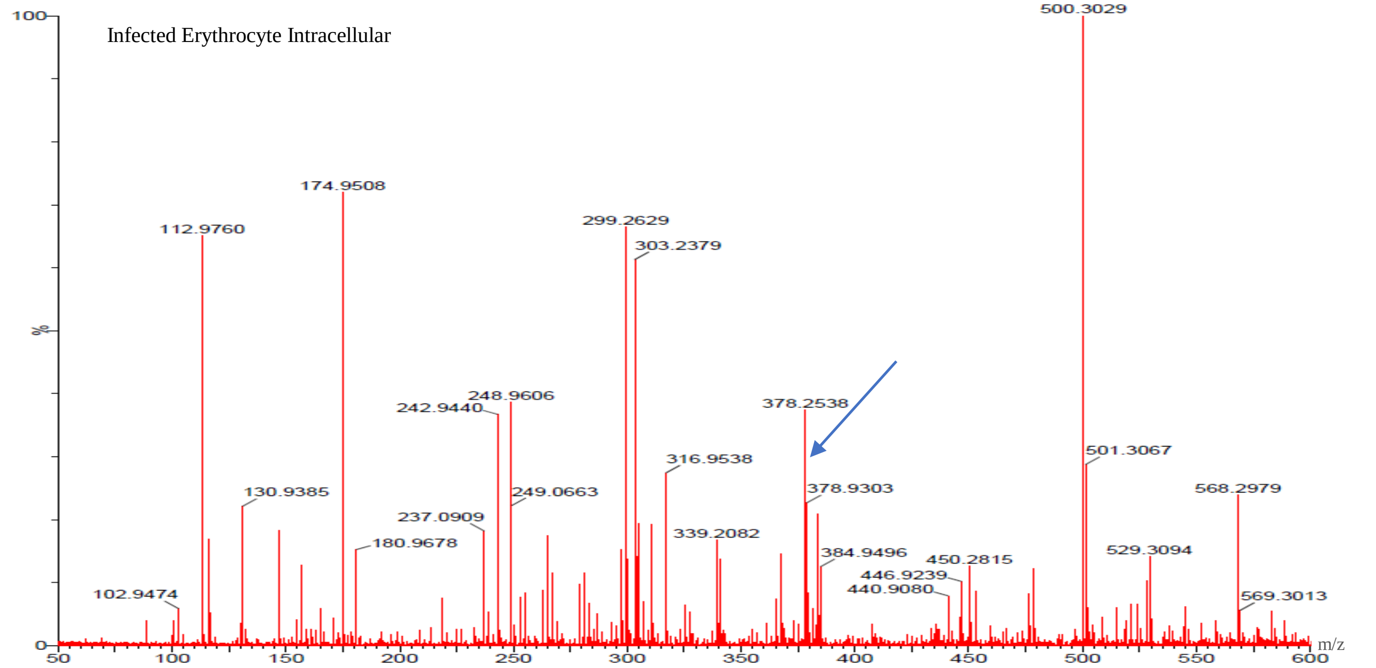
Infected Erythrocyte Intracellular
100
%
0
500.3029
174.9508
112.9760
299.2629
303.2379
248.9606
242.9440
378.2538
316.9538
501.3067
130.9385
249.0663
378.9303
568.2979
237.0909
339.2082
180.9678
529.3094
384.9496
450.2815
446.9239
440.9080
102.9474
569.3013
m/z
50
100
150
200
250
300
350
400
450
500
550
600

F)

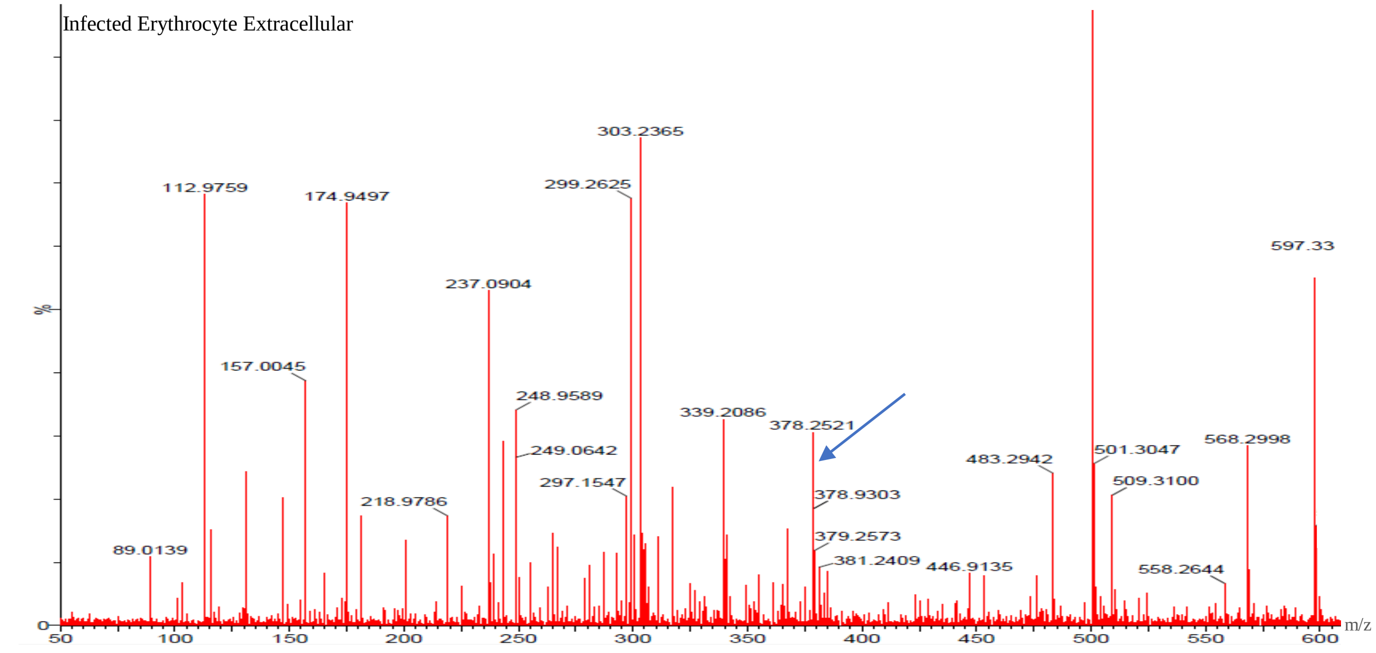
Infected Erythrocyte Extracellular
%
0
303.2365
112.9759
174.9497
299.2625
597.33
237.0904
157.0045
248.9589
339.2086
378.2521
249.0642
501.3047
568.2998
483.2942
297.1547
509.3100
218.9786
378.9303
379.2573
89.0139
381.2409
446.9135
558.2644
m/z
50
100
150
200
250
300
350
400
450
500
550
600

G)

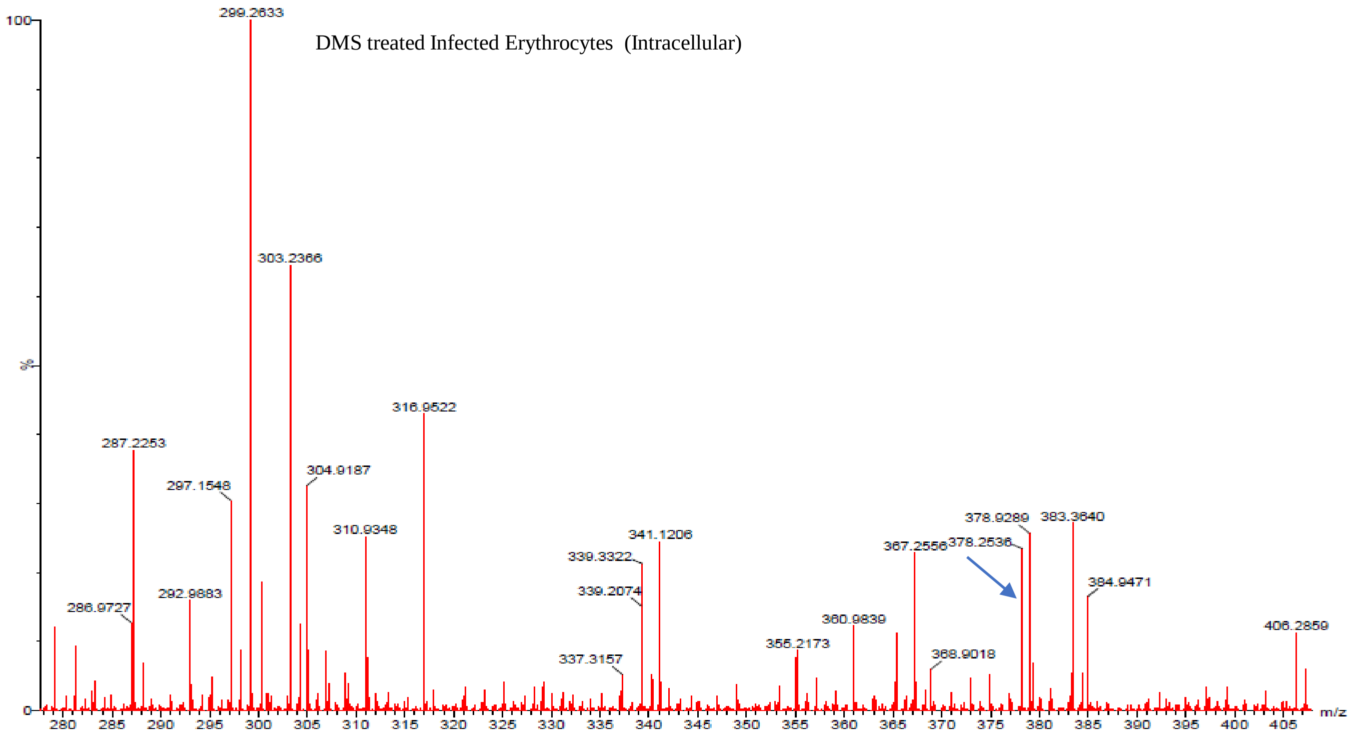

H)

Infected Erythrocyte Treated
Extracellular Not Detected(Because
of very low level)

Supplementary Figure 2. S1P detection in IC and EC milieu of uninfected erythrocytes and infected erythrocyte with DMS treatment and without treatment. Lipids were extracted from supernatant and lysed cells from uninfected erythrocytes and infected erythrocyte after DMS treatment and without treatment. The extracted lipids were subjected to LC/MS analysis for S1P detection. S1P characteristic peaks were detected at position 378.25 in the MS spectra for IC as well as EC milieu **(A-G)**. In **Figure 2 (H)** Due to lower level of S1P in DMS treated cells, peak was not detected.

A)

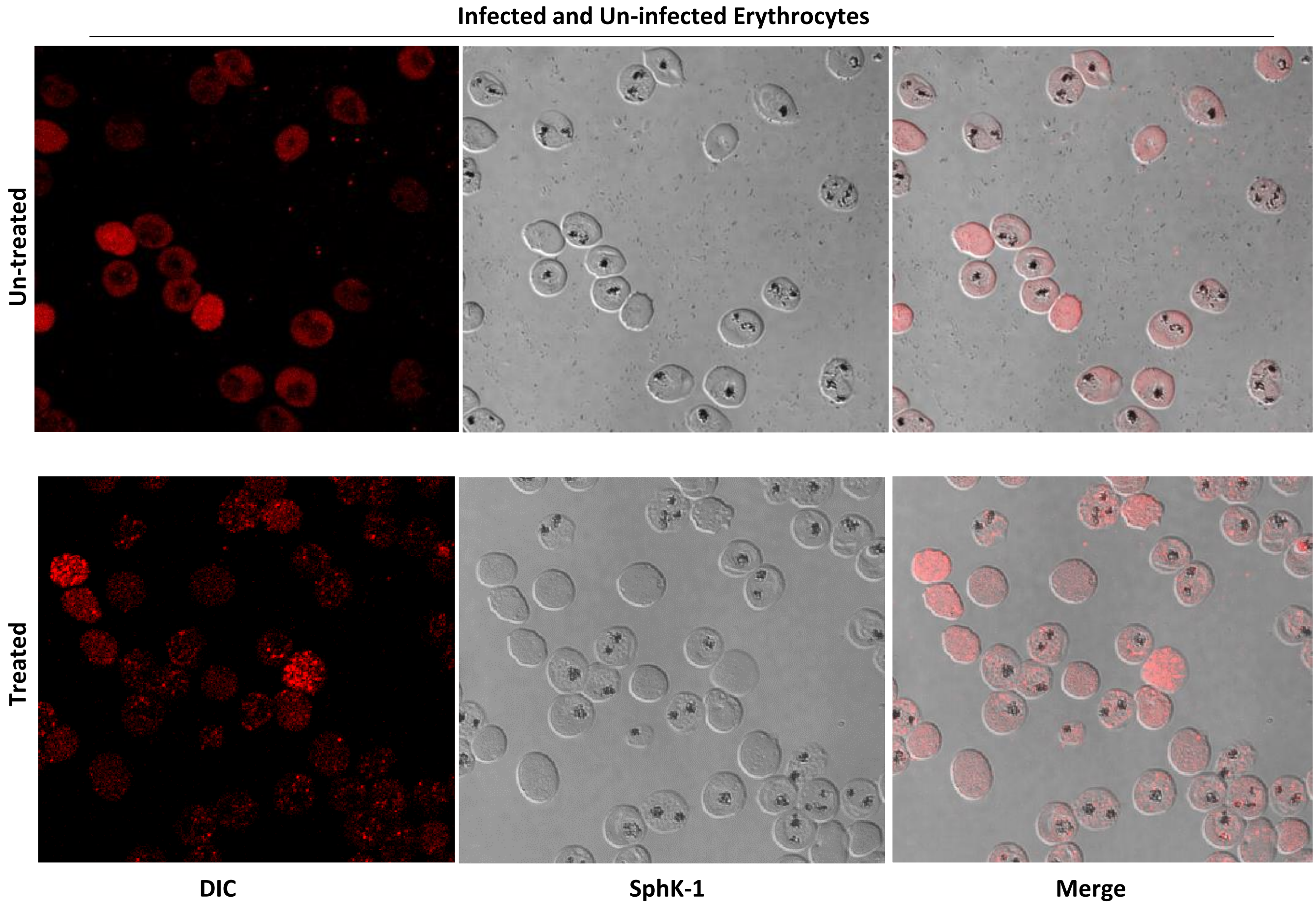

Supplementary Figure 3. Inhibition of host SphK-1 by specific inhibitor DMS causes decrease in SphK-1 protein level and phosphorylation. A) Confocal micrographs demonstrate altered host SphK-1 level in mixed-stage parasite culture following treatment with DMS (14 µM).

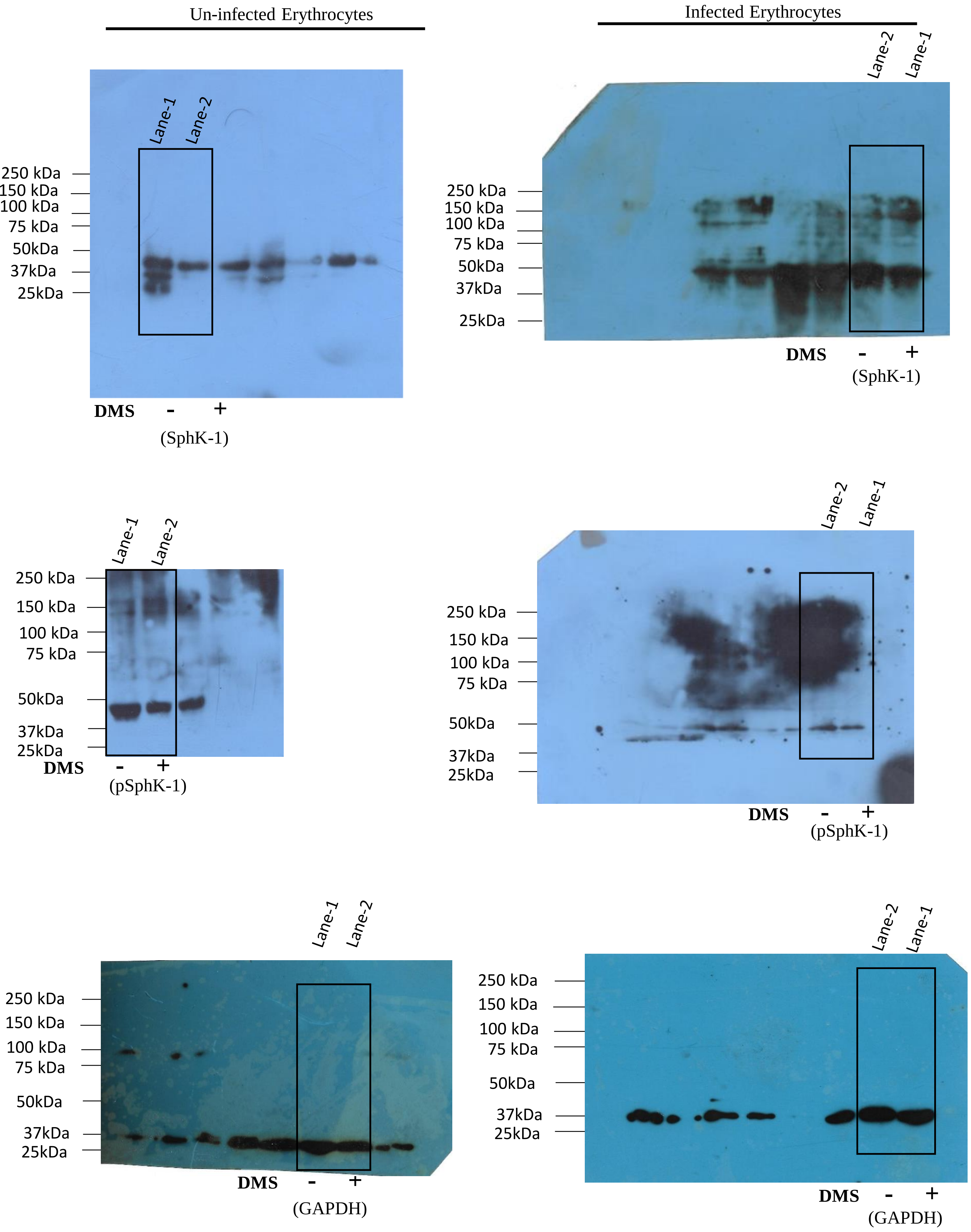

Supplementary Figure 4. Inhibition of host SphK-1 by specific inhibitor DMS causes decrease in SphK-1 protein level and phosphorylation. The phosphorylation status of SphK-1 and its level were evaluated by immunoblotting. Total cell lysates probed with GAPDH was used as a loading control.

A)

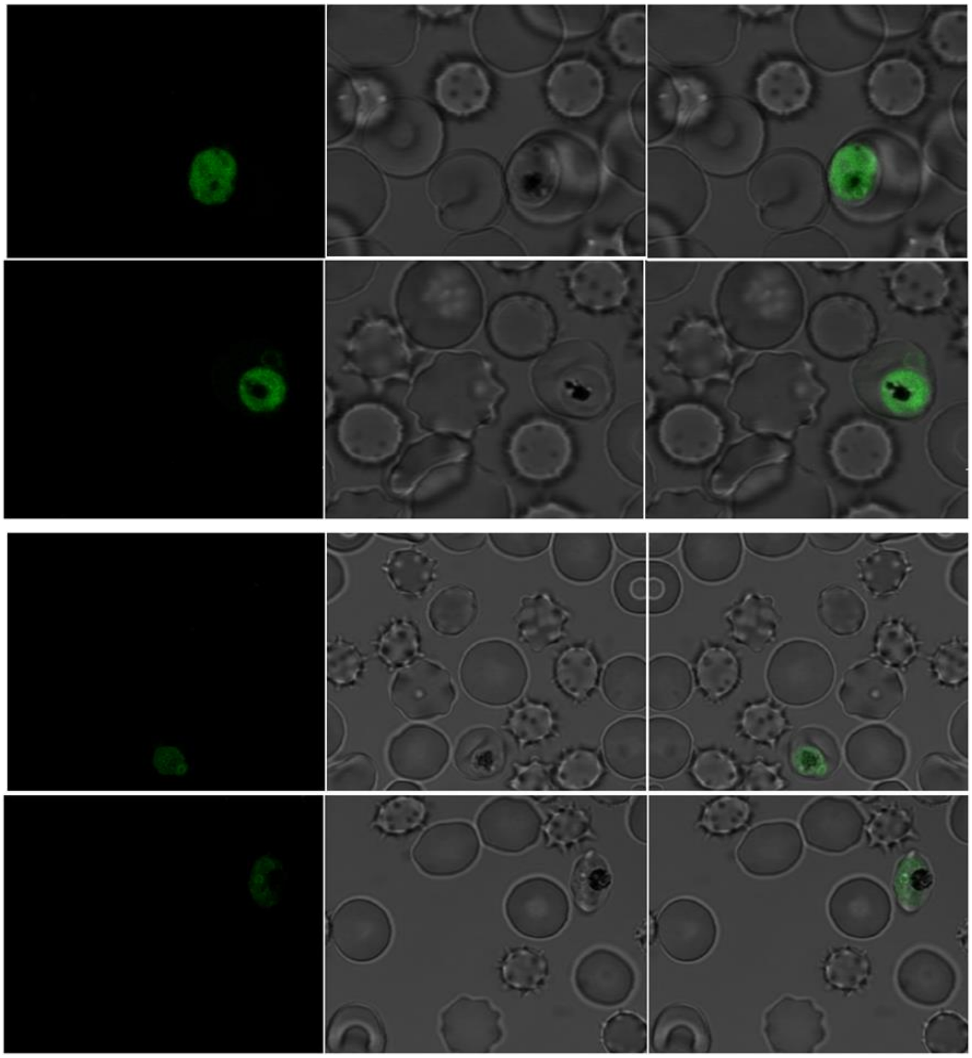

Supplementary Figure 5. Inhibition of host SphK-1 results in reduced glucose uptake. A) Evaluation of 2-NBDG uptake in parasite infected erythrocytes following DMS treatment by live-cell imaging. Respective MFI of individual infected erythrocytes were plotted against individual untreated cells.

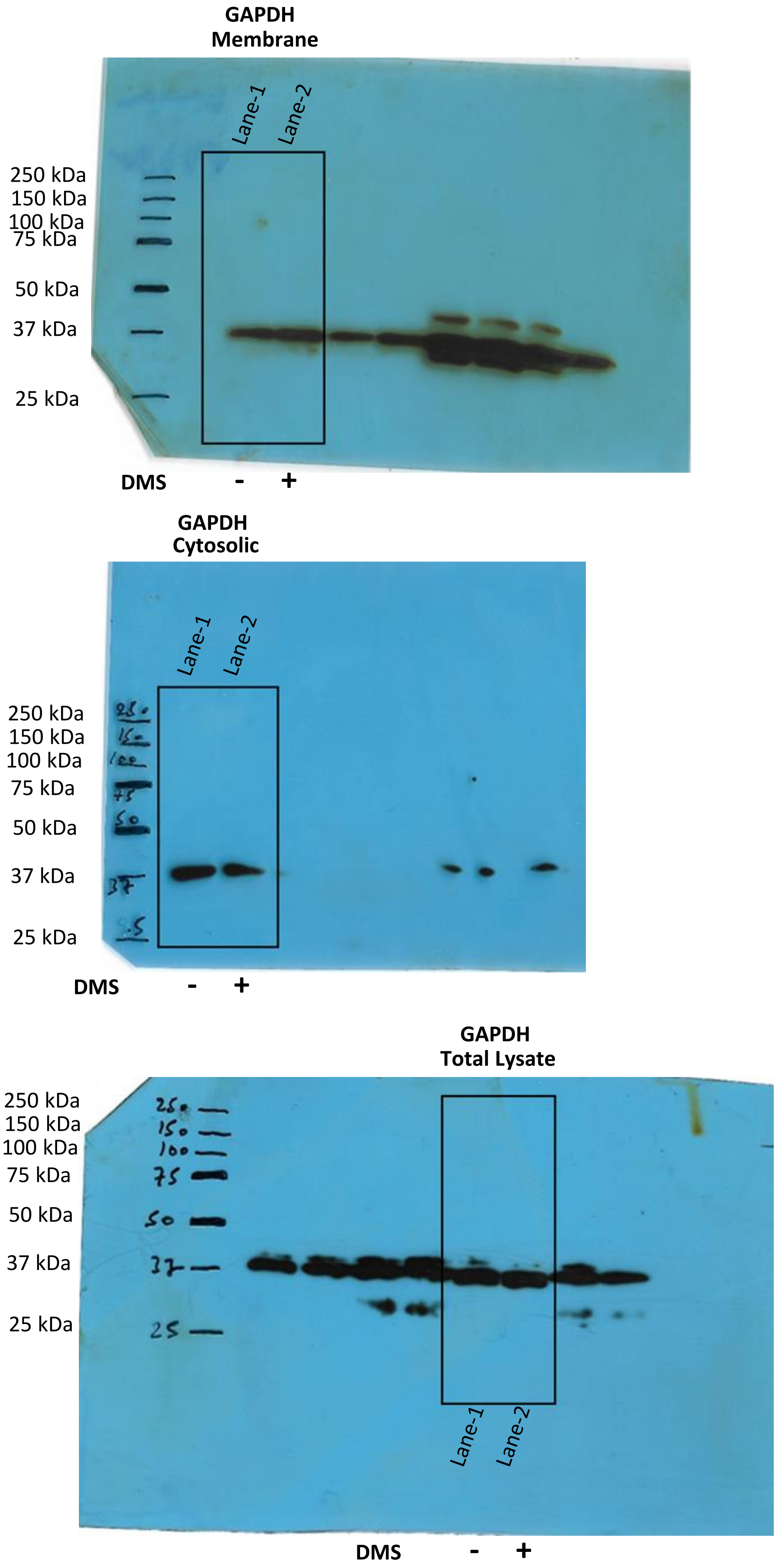

Supplementary Figure 6. Translocation of GAPDH in parasite infected erythrocytes upon host SphK-1 inhibition. Detection of GAPDH in both membrane and cytosolic fractions of DMS-treated infected erythrocytes**.** Total cell lysate was used as a loading control.
